# Impact of indocyanine green fluorescence angiography on surgeon action and anastomotic leak in colorectal resections. A systematic review and meta-analysis

**DOI:** 10.1007/s00464-025-11582-y

**Published:** 2025-02-03

**Authors:** Philip D. Mc Entee, Ashokkumar Singaravelu, Patrick A. Boland, Alice Moynihan, Ben Creavin, Ronan A. Cahill

**Affiliations:** 1 UCD Centre for Precision Surgery, UCD, Dublin, Ireland; 2 Department of Surgery, Mater Misericordiae University Hospital, 47 Eccles Street, Dublin 7, Dublin, Ireland

**Keywords:** Colorectal surgery, Rectal cancer, Anastomotic leak, Near-infrared laparoscopy, Indocyanine green

## Abstract

**Background:**

Indocyanine green fluorescence angiography (ICGFA) during colorectal surgery either reassures surgeons regarding intestinal perfusion sufficiency or prompts changed surgical strategy by indicating unsuspected insufficiency. This study describes existing evidence supporting ICGFA use in colorectal surgery, particularly regarding impact on intraoperative decisions.

**Methods:**

Searches were performed on PubMed, ScienceDirect, Scopus, Web of Science, Cochrane Collaboration databases on 5th December 2023, updated to 19th August 2024. Full English language publications of clinical studies in human patients undergoing colorectal resection with primary anastomosis with the use of ICGFA to assess bowel perfusion intraoperatively, with a control group, were included. Pooling of anastomotic leak (AL) rates was performed for primary outcome analysis with odds ratio (OR) and number-needed-to-treat (NNT) calculated regarding leak reduction.

**Results:**

45 studies comprising 14,333 patients were included, with 7 randomised controlled trials (2911 patients). Overall, AL rate was 6.8%, 4.5% with ICGFA and 8.5% without (OR:0.47, *p* < 0.001, NNT 23), increasing to 9.5%, 7.5% and 11.6%, respectively, in randomised controlled trials (OR:0.62, *p* < 0.01, NNT 25). In rectal resections, AL rate was 4.7% with ICGFA vs 11.5% without (OR: 0.38, *p* < 0.0001). 26 studies performed ICGFA before and after anastomosis formation and 19 used ICGFA only prior to bowel transection, with no significant difference of AL rate reduction on subgroup testing. ICGFA prompted a change in surgical plan in 8.4% of thirty-four studies reporting this. Interestingly, in these studies, leak rates overall were 3.7% when ICGFA matched surgeon judgement versus 5.7% when it prompted change (OR 0.51, *p* < 0.0025), versus 7.7% without (OR 0.45, *p* < 0.0001). In rectal resections, these figures were 5%, 8.8% (OR 0.42, *p* = 0.01) and 12.0% (OR0.39, *p* < 0.0001), respectively.

**Conclusions:**

ICGFA colorectal perfusion assessment is associated with lower anastomotic leak rates, especially when confirming surgeons’ judgement, and may so stratify patients post-operatively regarding subsequent anastomotic leak rate.

**Supplementary Information:**

The online version contains supplementary material available at 10.1007/s00464-025-11582-y.

Despite advances in surgical techniques and enhanced perioperative care, colorectal resections still carry significant risk of morbidity and mortality. Anastomotic leak (AL) is considered one of the more serious complications, being associated with increased rates of secondary complications and mortality, increased in-hospital stay and costs, and poorer oncological outcomes in survivors [1–7]. Reported AL rates vary between 4 and 26% [8–12] with patient-related factors including male sex, age, smoking, diabetes, obesity, neoadjuvant therapy and tumour location all contributing [13–16]. While the majority of these are fixed prior to surgery, there has been increasing attention regarding intraoperative tissue perfusion assessment to reduce incidence of AL [15, 17].

Indocyanine green fluorescence angiography (ICGFA) has been proposed as an efficient, real-time method of evaluating gastrointestinal anastomotic site perfusion [18, 19] with commercial laparoscopes now often including near-infrared (NIR) light visualisation of systemically administered ICG. Qualitative interpretation of ICGFA can then either reassure the surgeon’s judgement that perfusion is sufficient or encourage a change in transection site when selecting segments of intestine for anastomosis and/or check perfusion of the in situ anastomosis after construction (controlling so for any tissue tension impacting perfusion).

Many clinical studies have assessed the feasibility and possible effect on AL rates of ICGFA; however, their individual results and even meta-analyses to date have not confirmed the technique as standard of care [20–23]. While some randomised control trials (RCTs) have shown significantly lower AL rates associated with ICGFA use [24], others have not, [25] and other studies have either been too small or methodically inconsistent to convince the field of benefit. While further large RCTs are due to report soon [26, 27], a clear understanding of the current field will help understand, contextualise and integrate their findings as well as identify outstanding specific issues for consideration.

Therefore, we report the results of a systematic review and meta-analysis evaluating ICGFA and AL rates in colorectal resections overall and within important subgroups. Importantly, this study also assesses the impact of a change in surgical plan based on ICGFA findings regarding AL rates, as this is commonly perceived as the main mechanism of any AL reduction effect [28].

## Methods

### Registration

This systematic review was registered a priori in the International prospective register of systematic reviews “PROSPERO” (CRD42023484014).

### Search strategy

Systematic review was according to PRISMA checklist guidelines and recommendations. PubMed, ScienceDirect, Scopus, Web of Science and Cochrane Collaboration database were searched for relevant publications from inception to 5th December 2023 (updated to 19th August 2024) using the following Medical Subject Heading (MESH) search terms: “colorectal” (includes “colon” and “rectum”), “anastomosis”, “anastomotic leak”, “outcome”, “perfusion”, “fluorescence”, “angiography”, “indocyanine green”, “assessment”, “evaluation”. All terms were “exploded” to include subheadings and the truncation symbol (*) used as appropriate. The Boolean operator “OR” was used within concepts, while “AND” was used to link concepts. Titles and abstracts were then screened. Reference lists in relevant publications and Google Scholar were also screened for other relevant publications.

### Study selection

For inclusion in analysis, studies had to meet the following criteria:

- Full publication of a clinical study in patients undergoing colorectal resection with primary anastomosis for benign or malignant disease with outcome measure including AL rate.
- ICGFA use for assessment of bowel perfusion in patients with a control group without ICGFA.
- Full-text available in English.

Studies were excluded if any of the following criteria were met:

- No control group.
- No human patients (i.e. animal studies).
- Publication types such as case reports, letters to the editor, systematic reviews, meta-analyses, guidelines, conference abstracts.

### Outcomes of interest

The primary outcome was the rate of AL in patients both in whom ICGFA was and was not used. The main secondary outcome was the rate of change of surgical plan based on ICGFA interpretation and the rate of AL by operation, timing of ICGFA, as well as the rate of AL requiring intervention.

### Data extraction

Two reviewers (PMcE & AS) independently examined studies according to predefined strategy and criteria. Each extracted and recorded separately the title and publication details as well as population characteristics. In addition, if ICGFA was used, how was it used and on how many patients and, if documented, how its interpretation impacted intraoperative strategy (including effect on intended transection level before anastomosis or on anastomotic revision/defunctioning rates) was also recorded along with severity of AL and AL rate overall and for each group. Databases were reviewed by a third person (BC) and compared at the end of the reviewing process to limit selection bias, remove duplicates and clarify disparities.

### Statistical analysis

Statistical analysis was performed using RStudio 2023.09.1.494 [29]. The quality of included studies was assessed using the Newcastle–Ottawa Scale assessment tool. Risk of bias was assessed using the Rob 2 tool for RCTs and the ROBINS-I tool for other studies. Summary plots for RoB2 and ROBINS-I were created using the R package “robvis”. Traffic light plots were created using the “Shiny web app” [30]. Harbord’s score test for funnel plot asymmetry assessed publication bias. Statistical heterogeneity was determined by the *p* value of the Cochrane Q test and Inconsistency statistics (*I*^2^). In the absence of significant heterogeneity (*p* > 0.05), a common effects model was applied; otherwise, a random effects model was used. Cumulative AL rates were pooled. Meta-analysis used the Mantel–Haenszel method to calculate risk difference (RD) and odds ratio (OR) with a significance level of *p* < 0.05 and 95% confidence interval (CI). OR calculation using RStudio uses the Haldane-Anscombe correction when a cell contains a zero, which may impact OR with small n datasets. Number needed to treat (NNT) was calculated as the pooled risk difference reciprocal. Subgroup meta-analysis stratified by level of evidence (RCTs vs non-RCTs), site of operation, timing of ICGFA use and change of level data. Sensitivity analyses were performed to assess the robustness of the pooled results (for odds ratio and heterogeneity) by removing studies which were found to have a high risk of bias in our analysis. To model the impact of a change in surgical plan on leak rates, the lowest and highest possible values of leak rate in the change groups which resulted in a non-statistically significant difference of AL rate between the overall ICGFA (change and no change combined) and non-ICGFA groups were calculated at 95% confidence levels.

## Results

### Study, patient, operative and post-operative characteristics

**Fig. 1:**
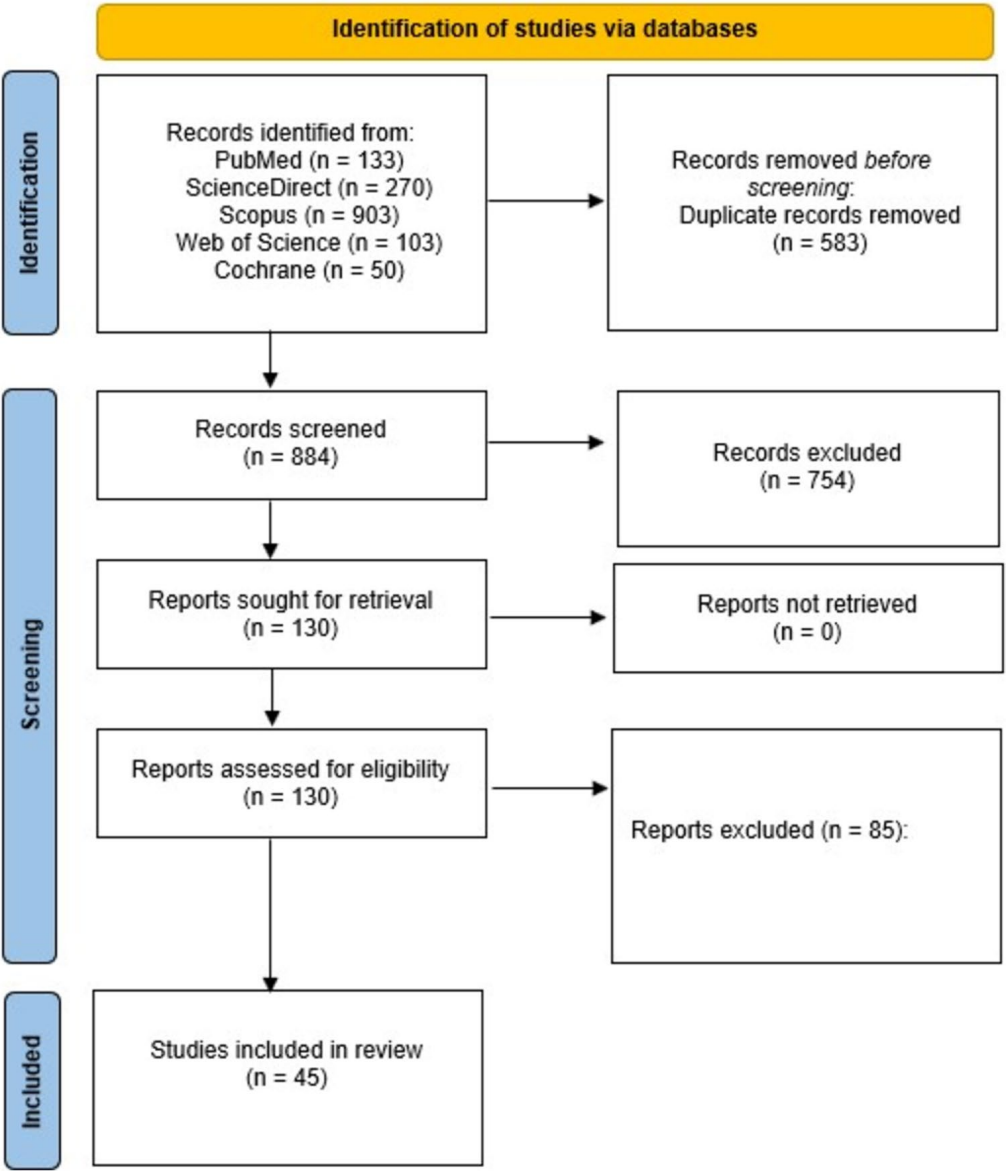
PRISMA flow chart for this systematic review regarding study search, selection and inclusion progress

After application of the selection criteria (Fig. 1), forty-five studies including 14,333 patients published between 2010 and 2024 were included. Thirty-one were retrospective cohort studies, seven were prospective cohort studies and seven were RCTs. Seventeen involved rectal operations only, eleven included left-sided and rectal procedures together and the remaining seventeen assessed a range of colorectal operations (Table 1 & Supplementary Table 1). In studies reporting on the prevalence of a diverting stoma (*n* = 34), the rates were 46.1% in both the ICGFA and control group (Supplementary Table 2).

**Table 1:** Included study and patient characteristics including first name author, study design, start date and duration, country of origin and patient number

Study	Design	Duration	Country	Number in study
Faber et al., 2024 [31]	RCT	Jul 20–Feb23	Netherlands	931
Eltaweel et al., 2024 [32]	RCT	Jan 22–Oct 22	Egypt	101
Qiu et al,. 2024 [33]	Retrospective	Jan 12–Jul 23	China	299
Gach et al., 2023 [34]	RCT	Dec 20–Aug 21	Poland	76
Watanabe et al., 2023 [28]	RCT, multicentre	Dec 18–Feb 21	Japan	839
Tueme-de la Peña et al., 2023 [35]	Retrospective	Jan 19–Sept 21	Mexico	168
Chen et al., 2023 [36]	Retrospective	Oct 19–Mar 22	China	286
Kondo et al., 2022 [37]	Retrospective	Jan 2014–Dec 2021	Japan	187
Neddermeyer et al., 2022 [38]	Retrospective	Jan 2017–Jan 2020	Germany	132
Losurdo et al., 2022 [39]	Retrospective	Jane 2015–Nov 2019	Italy	272
Hasegawa et al., 2022 [40]	Retrospective	May 2010–Aug 2017	Japan	169
Freund et al., 2021 [41]	Retrospective	2015–2021	USA	36
Chive et al., 2021 [42]	Retrospective	Jan 2003–June 2019	France	835
Aawsaj et al., 2021 [43]	Retrospective	Mar 2016–Oct 2019	England	127
Jafari et al., 2021 [25]	RCT, multicentre	Mar 2015–Feb 2017	USA	347
Kudszus et al., 2010 [15]	Retrospective	1998–2008	Germany	402
Yanagita et al., 2021 [44]	Retrospective	Oct 2011–Oct 2018	Japan	384
Benčurik et al., 2021 [45]	Prospective cohort	Aug 2015–Jan 2019	Czech Republic	200
Skrovina et al., 2020 [46]	Prospective cohort	Aug 2015-Feb 2017	Czech Republic	100
Marquardt et al., 2020 [47]	Retrospective	Jan 2014–Dec 2018	Germany	351
Su et al., 2020 [48]	Retrospective	Oct 2017–June 2019	China	189
Alekseev et al., 2020 [24]	RCT	Nov 2017–Aug 2019	Russia	377
Bonadio et al., 2020 [49]	Retrospective	Jan 2014–Jan 2017	Italy	66
De Nardi et al., 2020 [50]	RCT, multicentre	Jan 2016–Nov 2017	Italy	240
Impellizzeri et al., 2020 [51]	Retrospective	Nov 2014–Feb 2019	Italy	196
Ishii et al., 2020 [52]	Retrospective	Mar 2014–Dec 2018	Japan	488
Watanabe et al., 2020 [53]	Retrospective, multicentre	Sept 2014–Dec 2017	Japan	422
Tsang et al., 2020 [54]	Prospective cohort	Aug 2018–Sept 2019	China	131
Wojcik et al., 2020 [55]	Prospective cohort	June 2017–Dec 2018	France	111
Otero-Piñeiro et al., 2020 [56]	Retrospective	Nov 2011–June 2018	Spain	284
Spinelli et al., 2019 [57]	Retrospective, multicentre	2011–2017	Italy	64
Dinallo et al., 2019 [58]	Retrospective	June 2013–June 2016	USA	554
Shapera et al., 2019 [59]	Retrospective	Jan 2012–Apr 2018	USA	104
Boni et al., 2017 [60]	Prospective cohort	Oct 2014–Nov 2015	Italy	80
Kin et al., 2015 [61]	Retrospective	Nov 2005–Dec 2012	USA	346
Jafari et al., 2013 [62]	Retrospective	Feb 2011–Aug 2012	USA	38
Kim et al., 2017 [63]	Retrospective	July 2010–Mar 2016	South Korea	657
Wada et al., 2019 [64]	Retrospective	Jan 2009–May 2016	Japan	149
Ris et al., 2018 [65]	Prospective multicentre	Jan 2013–Dec 2016	Multicentre	1677
Mizrahi et al., 2018 [66]	Retrospective	2013–2016	USA	59
Foo et al., 2020 [67]	Retrospective	Jan 2013–Dec 2018	China	506
Flores-Rodriguez et al., 2023 [68]	Retrospective	Jan 2017–Dec 2020	Spain	785
Baset et al., 2022 [69]	Prospective cohort	Aug 2020–Feb 2022	Egypt	39
Brescia et al., 2018 [70]	Retrospective	Mar 2014–Apr 2017	Italy	182
Starker et al., 2018 [71]	Retrospective	Jan 2015–Apr 2016	USA	347

### Assessment of quality & bias

**Fig. 2:**
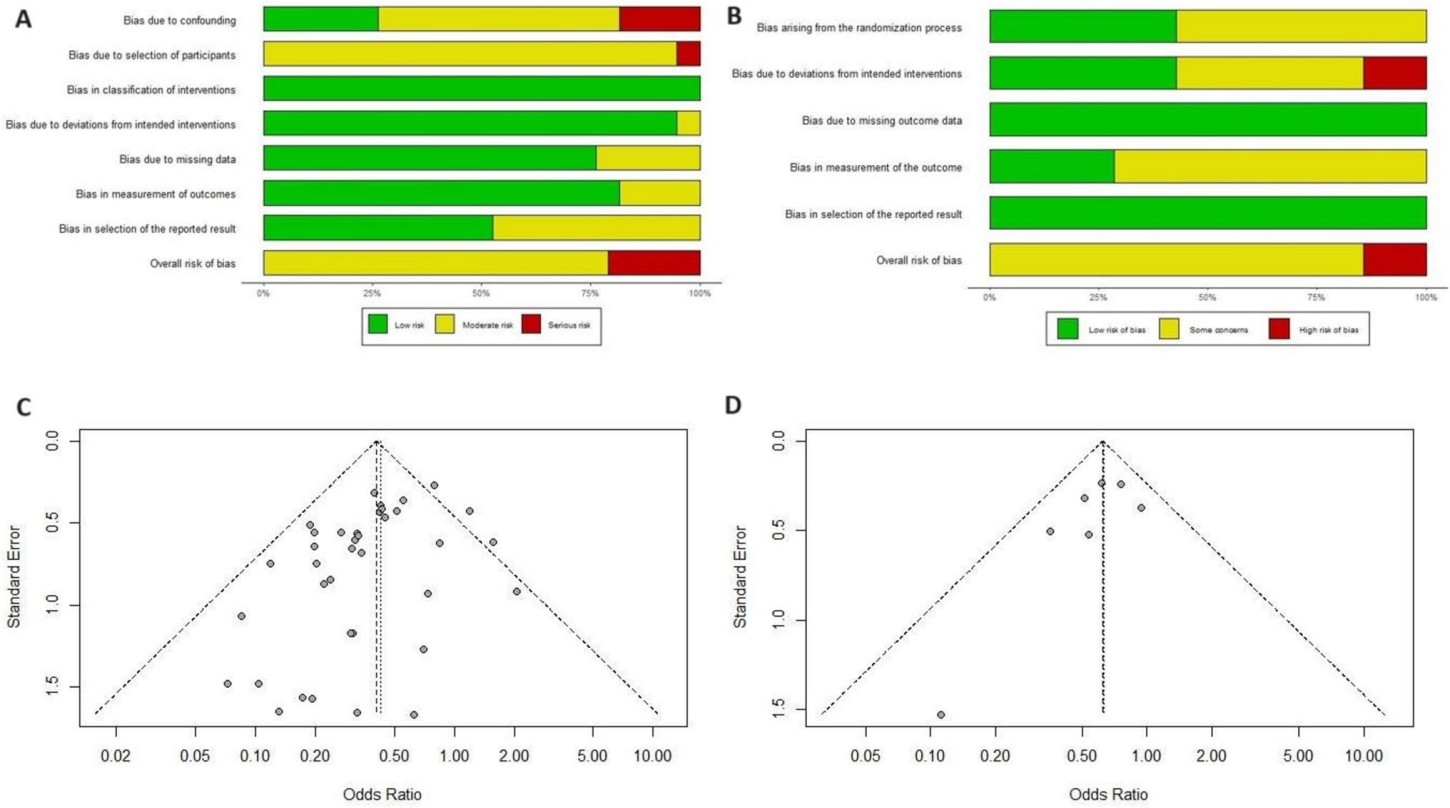
Summary/funnel plots regarding risk of basis of included studies (**A**) by ROBINS-1. (**B**) by RoB2 (**C**) included non-RCTs and (**D**) included RCTs. Asymmetry with Harbord’s score test yielding borderline *p* value (0.048) suggests potential publication bias in non-RCTs, whereas symmetry in RCTs indicates the absence of publication bias, although Harbord’s score test not performed here due to small sample size (*n* = 7)

The ROBINS-I tool found 30 of 38 non-randomised studies had moderate bias risk and eight had serious bias risk (i.e. highest risk of confounding and selection bias). The RoB 2 tool found one RCT at high risk of bias, with the other six having some concerns. For non-RCTs, funnel plot revealed asymmetry with Harbord’s score test yielding a *p* value (0.048), suggesting potential publication bias. For RCTs, funnel plot appears symmetrical indicating an absence of publication bias; Harbord’s score test was not performed because of small sample size (*n* = 7). (Fig. 2, Supplementary Figs. 1, 2).

### ICGFA use

**Fig. 3:**
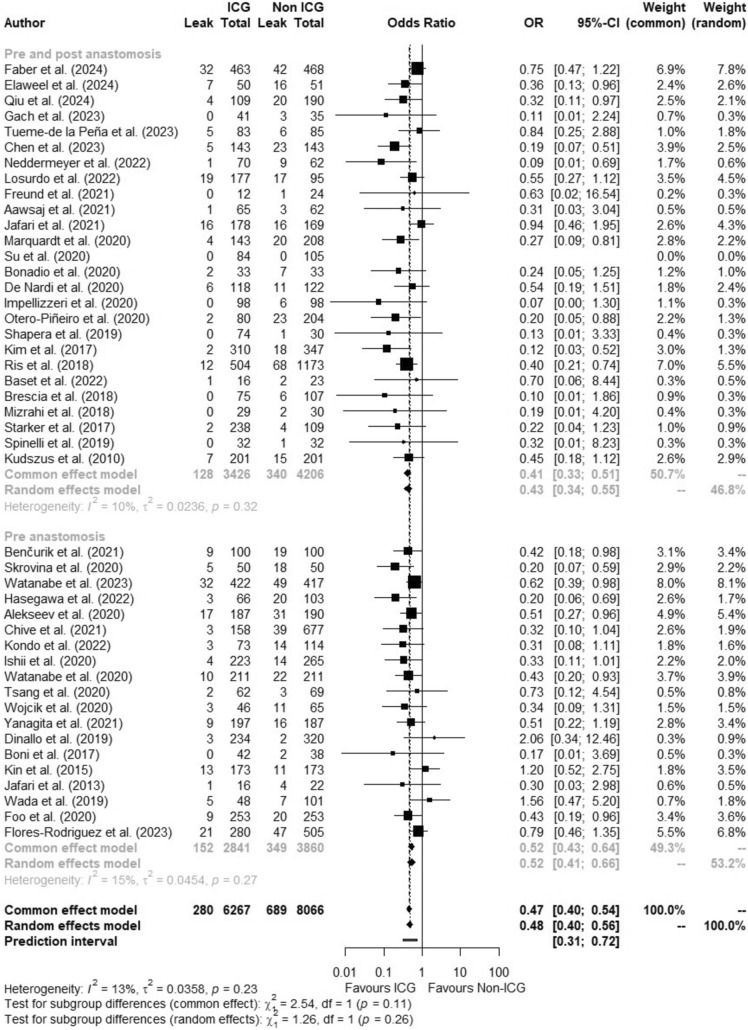
Forest plot showing odds ratio (OR) with 95% confidence interval (CI) of anastomotic leak in the ICGFA and non-ICGFA groups for studies where ICGFA was used before and after anastomosis formation versus when it was only used prior

In twenty-six studies (3426 patients), ICGFA was performed before bowel transection as well as after anastomosis formation, whereas it was performed only before bowel transection in nineteen studies (2841 patients). AL rates in studies assessing pre- and post-anastomosis were 3.7% for the ICGFA group and 8.1% for the non-ICGFA group (OR:0.41, 95%CI 0.33–0.51, *p* < 0.0001, *I*^2^ = 10.3%) while they were 5.4% and 9.0%, respectively, in studies only utilising ICGFA prior to anastomosis construction (OR:0.52, 95% CI 0.43–0.64, *p* < 0.0001, *I*^2^ = 15%). There was no significant difference on subgroup testing between these two groups (*p* = 0.11) (Fig. 3). After excluding studies with high risk of bias (*n* = 9), the OR for the former cohort was 0.43 (95%CI 0.33–0.57, *p* < 0.0001, *I*^2^ = 17.7%) and 0.54 (95%CI 0.44–0.67, *p* < 0.0001, *I*^2^ = 18.6%) for the latter.

### Overall AL rate

**Fig. 4:**
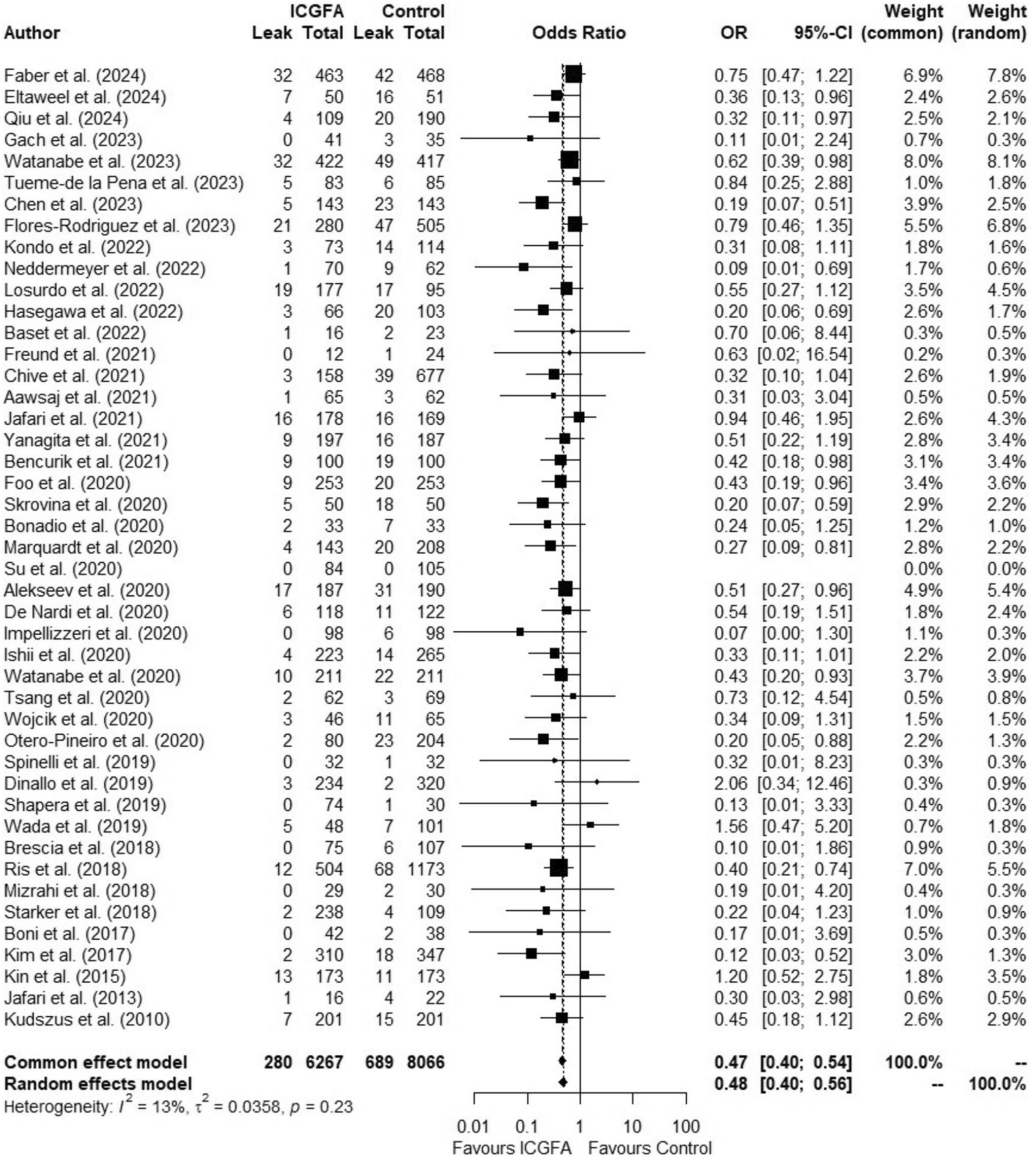
Forest plot showing odds ratio (OR) with 95% confidence interval (CI) of anastomotic leak in the ICGFA and non-ICGFA groups for all included studies

Of 14,333 patients, 6267 were in the ICGFA group and 8066 were controls. Overall, AL rate was 6.8% (*n* = 969). Pooled AL incidence in patients receiving ICGFA was 4.5% compared with 8.5% in controls (OR:0.47, 95%CI 0.40–0.54, *p* < 0.0001, *I*^2^ = 13%) (Fig. 4). Pooled meta-analysis of all studies revealed a risk difference of −0.0451 (95%CI −0.0533, −0.0370, *I*^2^ = 51.2%, *p* < 0.0001) and a NNT of 23 (95%CI 18.16–30.90). After excluding studies with a high risk of bias (*n* = 9), the OR was 0.50 (95%CI 0.42–0.58, *p* < 0.0001, *I*^2^ = 17.2%). When considering studies where data were reported on the severity of AL (*n* = 28), the pooled incidence of AL requiring intervention (defined as Grade B/C [11] or ≥ Clavien-Dindo Grade III) in patients receiving ICGFA was 4% compared with 8.1% in controls (OR:0.48, 95%CI 0.38–0.60, *p* < 0.0001, *I*^2^ = 10.6%)(Supplementary Fig. 3). After excluding studies with a high risk of bias (*n* = 2), the OR was 0.50 (95%CI 0.39–0.63, *p* < 0.0001, *I*^2^ = 11.1%).

### Subgroup AL rate analysis by level of evidence

**Fig. 5:**
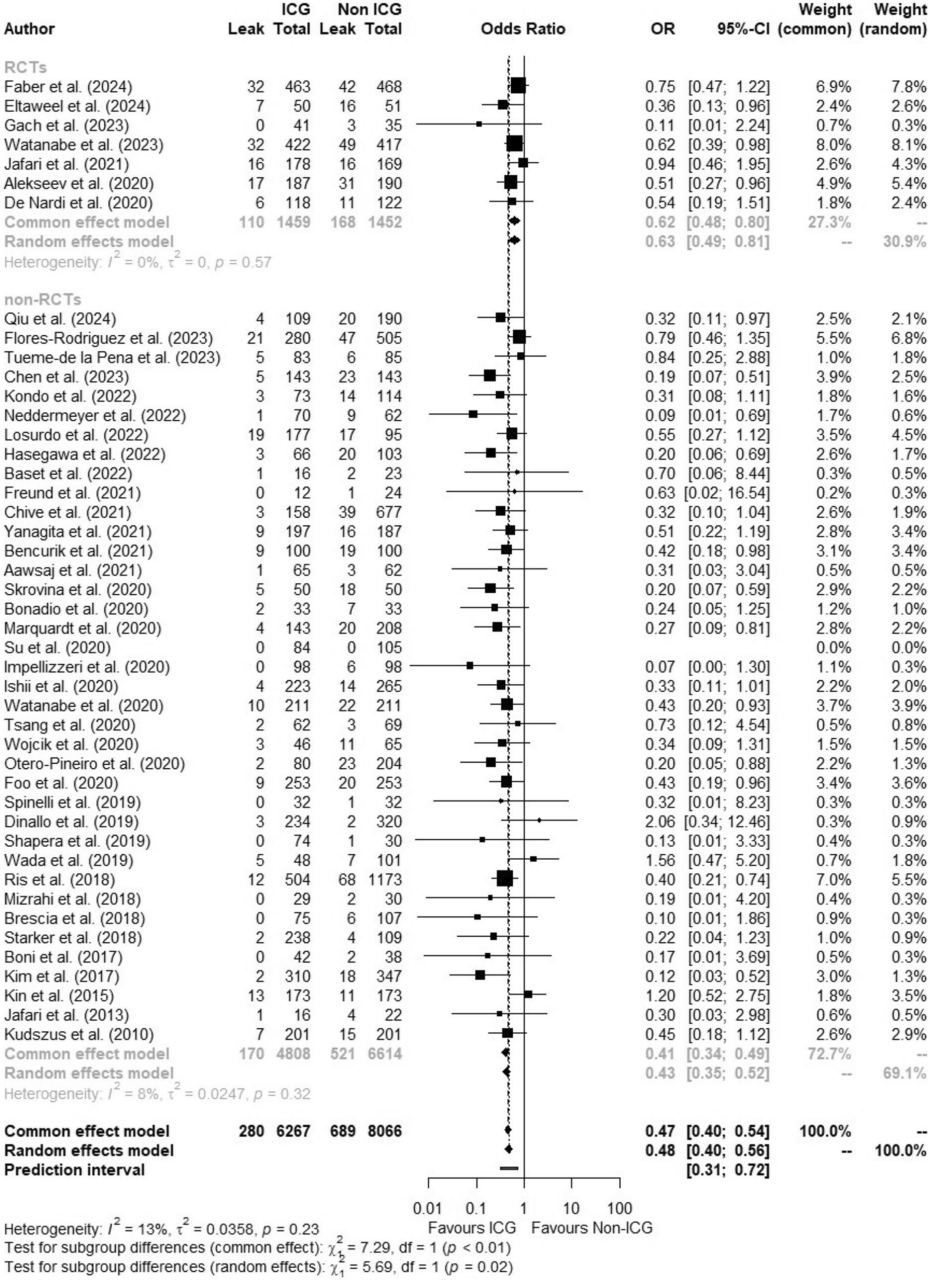
Forest plot of subgroup meta-analysis showing odds ratio (OR) with 95% confidence interval (CI) of anastomotic leak in the ICGFA and non-ICGFA groups by level of evidence

In non-RCTs (38 studies), AL rates overall were 6.0%, 3.5% in those receiving ICGFA (170/4808 patients) compared to 7.9% (521/6614 patients) in controls (OR:0.41, 95%CI 0.34–0.49, *p* < 0.0001, *I*^2^ = 8.5%). In RCTs (7 studies), the AL rate overall was 9.5%, 7.5% in ICGFA group (110/1459 patients) compared to 11.6% (168/1452 patients) in controls (OR:0.62, 95%CI 0.48–0.80, *p* < 0.01, *I*^2^ = 0%). Subgroup differences were significant (*p* < 0.01) (Fig. 5). NNT was 22 in non-RCTs (95%CI [17.40–31.32], RD −0.0447, 95%CI [−0.0575; −0.0319], *p* < 0.0001, *I*^2^ = 55.5%) and 25 in RCTs (95%CI [16.3–52.9], RD −0.0401, 95%CI [−0.0613; −0.0189], *p* < 0.01, *I*^2^ = 11.1%). After excluding non-RCT studies with a high risk of bias, the OR was 0.43 (95%CI 0.35.0.53, *p* < 0.0001, *I*^2^ = 15%), and after excluding the 1 RCT with a high risk of bias, the OR was 0.63 (95%CI 0.49–0.82, *p* < 0.001, *I*^2^ = 0%).

### Subgroup AL rate analysis by site of procedure

**Fig. 6:**
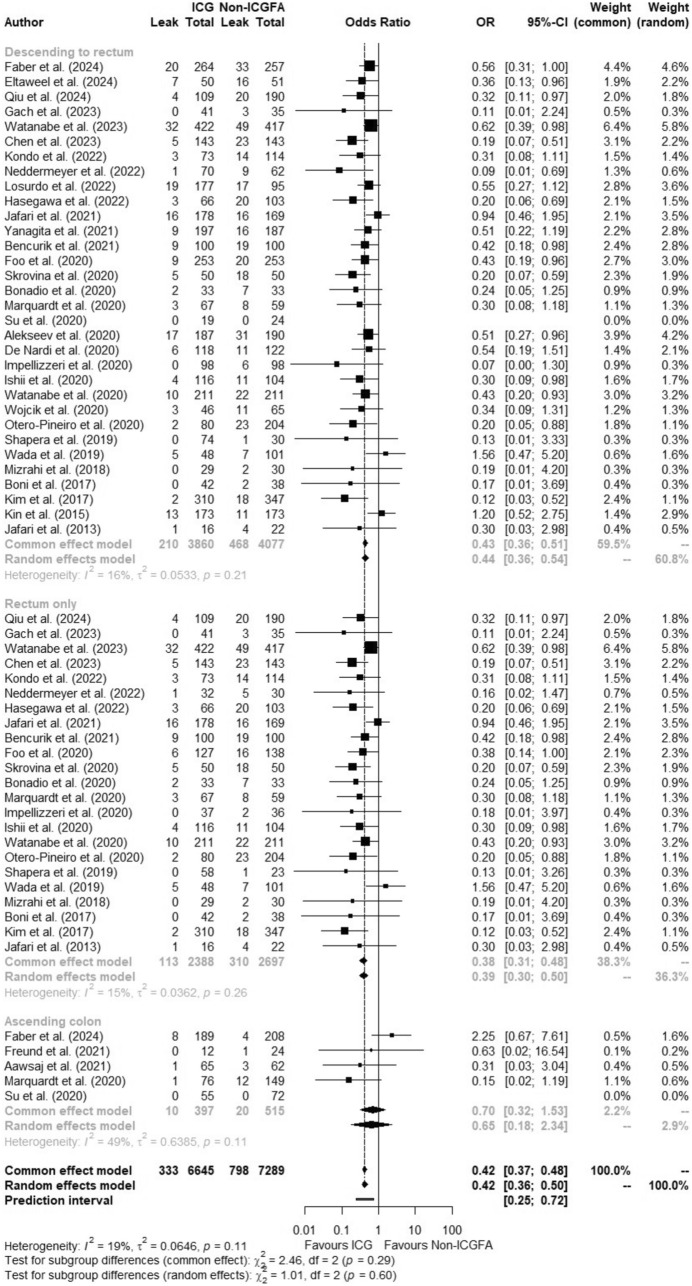
Forest plot of subgroup meta-analysis showing odds ratio (OR) with 95% confidence interval (CI) of anastomotic leak in the ICGFA and non-ICGFA groups by site of procedure

AL rates with ICGFA in patients who underwent left-sided resections (left hemicolectomy, sigmoid colectomy and rectal resection, not Hartmann’s reversals, 7937 patients) were 5.4% versus 11.5% without (OR:0.43, 95%CI 0.36–0.51, *p* < 0.01, *I*^2^ = 16.4%). After excluding studies with a high risk of bias (*n* = 4), the OR was 0.45 (95%CI 0.37–0.55, *p* < 0.01, *I*^2^ = 21.5%). In studies involving only patients undergoing rectal resection (5085 patients from 18 studies reporting rectal resections alone with extracted numbers from five other studies), the rate of AL was 4.7% with ICGFA compared to 11.5% without (OR:0.38, 95%CI 0.31–0.48, *p* < 0.0001, *I*^2^ = 14.5%). NNT according to level 1 evidence was 29 (95%CI [15.10–437.30], RD −0.03, 95%CI [−0.066; −0.002], *p* = 0.03, *I*^2^ = 0%). After excluding studies with a high risk of bias (*n* = 3), the OR was 0.41 (95%CI 0.32–0.52, *p* < 0.0001, *I*^2^ = 18.9%). For patients undergoing right-sided operations (912 patients), AL rate was 2.5% (10/397 patients) with ICGFA and 3.9% (20/515 patients) without (OR:0.70, 95%CI 0.32–1.53, *p* = 0.37, *I*^2^ = 49.5%) (Fig. 6).

### Subgroup AL rates, impact of change in surgical plan due ICGFA

The rate of change of surgical plan with ICGFA was 8.4% (385/4570 patients) in the thirty-four studies that recorded this with associated AL rates. In 354 of these patients (92%), change was to the level of transection before anastomotic construction. Of the 2115 patients in these studies for whom ICGFA was repeated after anastomosis formation with AL rates reported, 10 patients underwent a specified change of surgical plan (no stoma where one had initially been planned in five patients, four patients underwent anastomosis revision and one had additional splenic flexure mobilisation, with none of these patients suffering AL). In twenty-one patients, the exact change of plan was not specified, one of whom suffered AL.

**Fig. 7:**
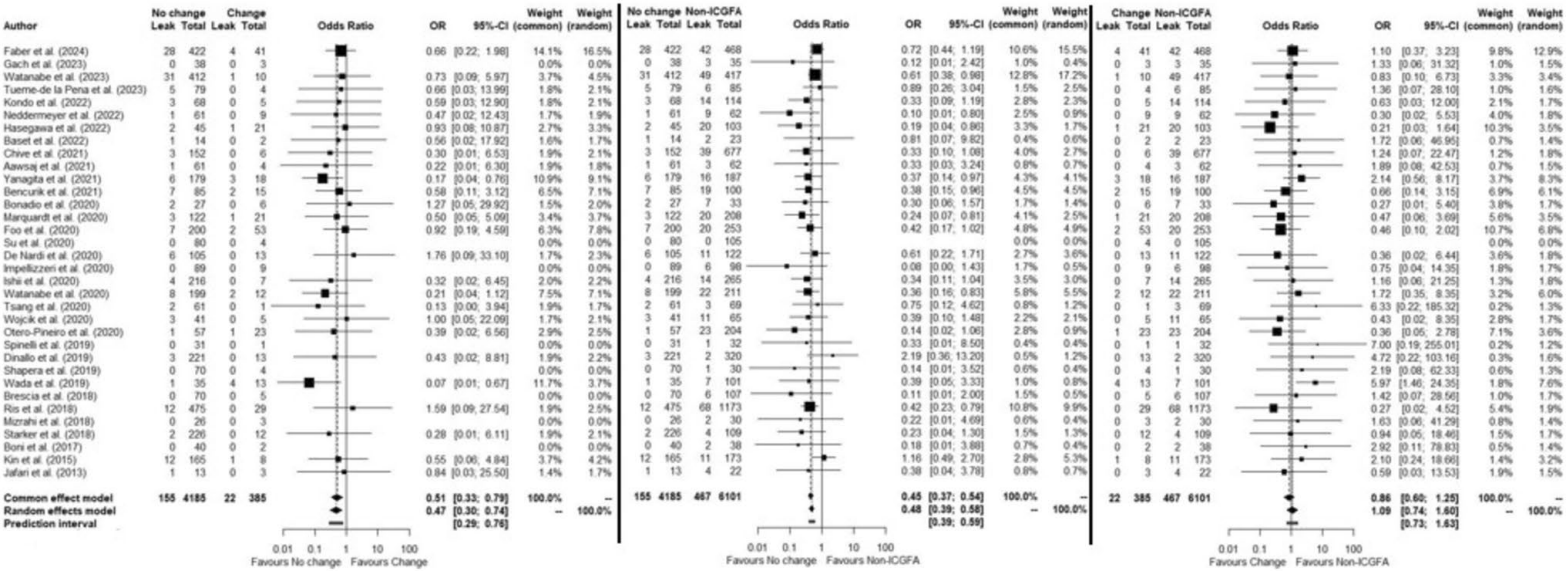
Forest plot of subgroup meta-analysis in no change, change and non-ICGFA groups – overall

AL rates in these studies with change of surgical plan data were 3.7%, 5.7% and 7.7% for no change (ICGFA matched surgeon judgement), change (ICGFA discordant with surgeon judgement and action resulted) and no ICGFA, respectively. AL rate when ICGFA matched surgeon judgement was significantly lower than those in whom ICGFA induced a change (OR:0.51, 95%CI 0.33–0.79, *p* < 0.0025, *I*^2^ = 0%) and significantly lower than those not receiving ICGFA (OR:0.45, 95%CI 0.37–0.54, *p* < 0.0001, *I*^2^ = 0%). AL rates in those in whom ICGFA induced a change in surgical plan were not significantly lower than those not receiving ICGFA (OR:0.86, 95%CI 0.60–1.25, *p* = 0.44, *I*^2^ = 0%) (Fig. 7). After excluding studies with a high risk of bias (*n* = 5), the OR figures were 0.50 (95%CI 0.32–0.78, *p* < 0.0025, *I*^2^ = 0%), 0.48 (95%CI 0.39–0.59, *p* < 0.0001, *I*^2^ = 0%) and 0.90 (95%CI 0.61–1.33, *p* = 0.60, *I*^2^ = 0%), respectively.

**Fig. 8:**
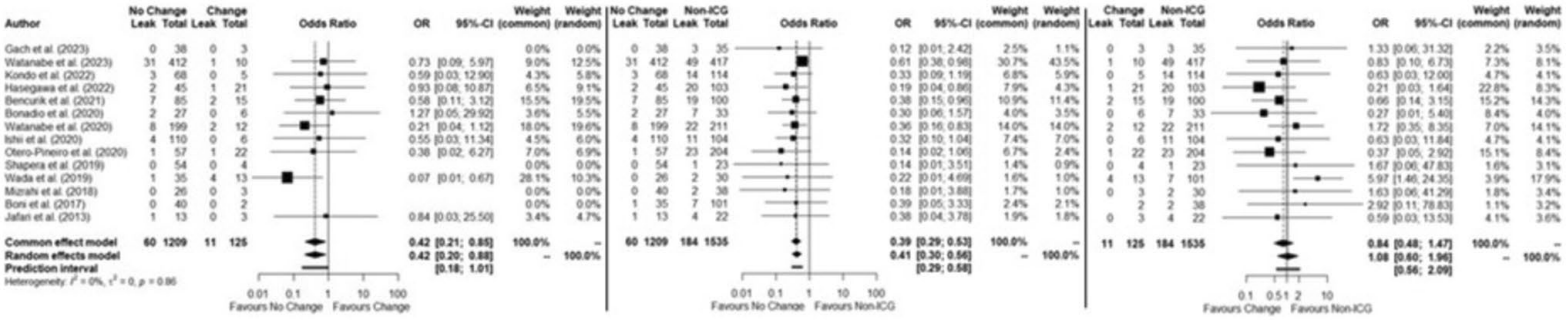
Forest plot of subgroup meta-analysis in no change, change groups and non-ICGFA groups—rectal resections

In the subgroup undergoing rectal resection with change of surgical plan data (all whose changes involved a change in the intended transection line), AL rates were 5%, 8.8% and 12.0% for no change, change and no ICGFA, respectively. Again, AL rate was significantly lower when ICGFA did not change the transection site versus when it did (OR:0.42, 95%CI 0.21–0.85, *p* = 0.01, *I*^2^ = 0%) and versus those without ICGFA (OR:0.39, 95%CI 0.29–0.53, *p* < 0.0001, *I*^2^ = 0%). Also again, AL rates when ICGFA induced a change of plan were not significantly lower than those without ICGFA (OR:0.84, 95%CI 0.48–1.47, *p* = 0.54, *I*^2^ = 0%) (Fig. 8).

In the four RCTs where change of mind and accompanying AL data were reported, AL rates were 6.7% (65/977), 7.5% (5/67) and 10.1% (105/1042) for no change, change and no ICGFA, respectively. AL rate was not significantly lower when ICGFA did not change the transection site versus when it did (OR:0.77, 95%CI 0.31–1.93, *p* = 0.58, *I*^2^ = 0%). AL rate was significantly lower when ICGFA did not change the transection site compared to those without ICGFA (OR:0.64 95%CI 0.46–0.88, *p* < 0.01, *I*^2^ = 0%) but not significantly different between those in whom there was a change of plan compared to those without ICGFA (OR0.91, 95%CI 0.38–2.17, *p* = 0.83, *I*^2^ = 0%) (Supplementary Fig. 4).

In the two RCTs in this group only reporting on rectal operations, the AL rates were 6.9% (31/450), 7.7% (1/13) and 11.5% (52/452) for no change, change and no ICGFA, respectively. There was no significant difference when ICGFA did not change the surgical plan versus when it did (OR:0.73, 95%CI 0.09–5.97, *p* = 0.77, *I*^2^ = NA); however, AL rate here was also significantly lower in the no change group versus no ICGFA (OR:0.57, 95%CI 0.36–0.91, *p* = 0.02, *I*^2^ = 9%), but not when ICGFA did change the surgical plan versus those without ICGFA (OR:0.95, 95%CI 0.17–5.36, *p* = 0.95, *I*^2^ = 0%) (Supplementary Fig. 5).

**Fig. 9:**
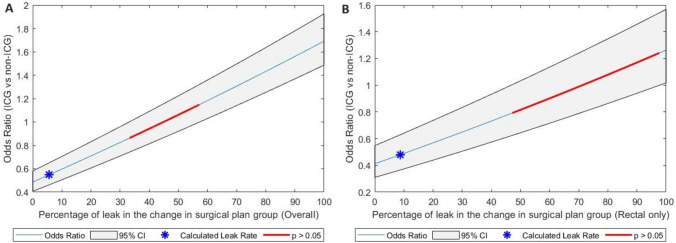
Extrapolated percentage rate of leaks otherwise expected in change of surgical strategy group (**A**) overall and (**B**) in rectal resections alone

To examine further for a mechanistic effect underlying the significant difference in AL rates between the intervention and control groups, the impact of a change in surgical plan on an individual basis was analysed. From the figures reported, it can be extrapolated that, for a non-significantly different leak rate between the intervention and control groups overall, those patients who had a change in their surgical plan due to ICGFA would otherwise have been expected to suffer AL at a rate of 33.2–57.1% overall (all studies) or at a rate of 47.2–97.6% in the rectal resection only group (Fig. 9). Considering RCTs alone, the equivalent figures are 14.9–94% overall and 30.8–100% for rectal only RCTs (Supplementary Fig. 6).

## Discussion

Given the detrimental effect of AL on both patients and healthcare systems [72], many strategies such as antimicrobial prophylaxis and bowel preparation, anastomotic stapling and perianastomotic drain placement have been investigated in an attempt to reduce AL rates [73–75]. With tissue perfusion being considered a significant factor in anastomotic healing, with an inferred effect on AL rates [3, 15], different intraoperative measurement techniques have been proposed including doppler ultrasound, laser Doppler flowmetry and tissue oxygen spectroscopy [76–78]; however, these techniques have not been generally adopted. In contrast, ICGFA has developed a wide user base [79] since Kudzsuz et al. [15] became the first group to report a benefit, with many major surgical equipment manufacturers now including this capability in their laparoscopic and robotic-assisted surgery stacks. While many clinical studies have now been performed, there have been inconsistencies in their findings, especially early on, with some studies even reporting a non-significant increased rate of AL with ICGFA use [58, 61, 64]. As many studies have been relatively small and non-randomized, aggregation is needed to better understand the true effect of ICGFA and now meta-analyses are indicating lower odds of AL with ICGFA overall [20–23].

This updated meta-analysis also indicates that ICGFA use is associated with a significantly lower rate of AL overall, albeit less so in RCTs. The majority of published studies have assessed left-sided resections (left hemicolectomy, sigmoid colectomy and rectal resection), likely because left-sided resections are associated with higher rates of AL than right-sided operations, [80] and so there is greater potential benefit from any intervention which may reduce AL rates (and powered studies to prove benefit need lower numbers). This assertion is supported by the findings of our analysis where the AL rate for right-sided resections was 2.5% with ICGFA and 3.9% without compared to 5.4% and 11.5%, respectively, for left-sided resections. Statistically significant lower AL rates were seen with ICGFA in these procedures overall, when only considering AL requiring intervention, and indeed in rectal procedures alone, in both RCTs and non-RCTS. Two additional major randomised trials have concluded recruitment late last year and are expected to report this year, INTACT [26] (836 patients all having rectal cancer resections) and ICG-COLORAL [27] (840 patients). These studies, on publication, will add another 1676 patients to the evidence base strengthening the level one evidence.

The benefit of ICGFA in colorectal surgery is assumed to be that it allows visualisation of the microcirculation of selected segments of bowel intended for or involved in the anastomosis and so informs regarding any perfusion insufficiency that might otherwise contribute to a malperfusion-associated AL [62] at a time it can be corrected (i.e. intraoperatively). This additional check is most commonly performed before rather than only after anastomosis construction, with many studies only using it before. However, our results indicate no significant difference in AL rates when ICGFA is used before and after anastomosis formation compared to when used only before bowel transection. If the surgeon finds the intended transection point or formed anastomosis is poorly perfused on ICGFA, they alter their surgical plan. Importantly, however, patients with ICGFA confirmation of the surgeon’s own assessment have the lowest leak rate. There is additional valuable information in this that could be factored into perioperative decisions such as whether the patient may be commenced on an Early Recovery After Surgery pathway, be considered for early discharge or perhaps forego intraoperative defunctioning stoma. This cohort of patients has a reassuringly lower AL rate of 3.7%; however, this is still a significant risk given the potential for leak-related morbidity and mortality. A reassuring ICGFA assessment should therefore be considered as one of many factors which may influence perioperative decisions. Conversely, those in whom ICGFA identified worse perfusion than the surgeon estimated have an overall AL rate (including in rectal resections alone) that is not significantly different to patients not having ICGFA, even when corrective action has been taken. This means caution is needed regarding these patients post-operatively. One would expect that changing the transection point to a segment of bowel with better perfusion as indicated by the ICGFA signal would reduce the leak rate below that of the control group; however, we have shown that in the cohort where a change has been made based on the ICGFA interpretation, they have an AL rate that is equal to that of the control group. We hypothesise that ICGFA in this change cohort does two things: 1. It identifies patients who are at higher risk of AL; 2. It reduces the leak rate of this high-risk cohort to that of the control group, leading to a reduced rate of AL overall for the intervention group compared to the control group. These patients may be suffering a global perfusion deficit that is not fully addressable by a “simple” change to another intestinal segment for anastomosis. Alternatively, moving the transection line more proximally may result in more tension to the anastomosis, in turn increasing the relative risk of AL versus those in whom no change was performed (this point should encourage ICGFA use again after anastomotic construction). This hypothesis may be supported by the fact that in the 385 cases where a change in surgical plan was recorded, despite the transection line being moved more proximally in 92% of cases, additional splenic flexure mobilisation was only reported in one case. Surgeons may consider a different pathway for these patients post-operatively, including moving the patient off enhanced recovery pathways into high dependency monitoring, early post-operative imaging or even defunctioning or no anastomosis.

The ICGFA benefit is most easily understood, if it is derived from the change in surgical plan, with the assumption that this group would otherwise be destined for an ischaemic leak. However, while likely, this is not necessarily true as can be seen by the extrapolated range of rates that could statistically be expected (conversely even sufficient ICGFA does not eliminate all risk of AL, meaning other factors contribute). It is possible that some other factors associated with ICGFA may have small additional effects such as some intangible effect on the focus of both the surgical and anaesthetic teams in effecting a “slow down” moment around anastomotic construction, that may influence some technical aspects such as stapler placement and/or temporal relief of vascular spasm after stapling. In addition, or alternatively, the ICGFA signal may encourage the surgeon to place the stapler directly into the “green zone” indicated by the ICGFA proximal to the perfused/non perfused interface (even slightly proximal to the mesocolic cut line) avoiding any area of local spasm/stapler impact. Other considerations, while speculative, include a therapeutic benefit of NIR alone or in association with ICG potentially via a phototherapeutic bystander effect, or other immunological impact (including on the local microbiome), although exposure times are low making this unlikely.

Nonetheless, the finding of reduced AL overall with ICGFA is clear from the available evidence base. While the upcoming RCTs may further strengthen its case, other concerns beyond its effectiveness may need consideration. The absolute effect as indicated by NNT may be perceived as high, meaning that, along with the view that AL is a multifactorial event, surgeons may reserve ICGFA for only perceived high-risk cases (conversely, understanding ICGFA value in identifying those with lowest leak risk strengthens the argument for routine use). Some additional considerations impacting ICGFA uptake concern availability of ICG which differs in different jurisdictions and lack of standardised ICGFA training pathways (which may contribute to interobserver variation in interpretation [81]), along with the added cognitive burden associated with the additional intraoperative step in what is an already complex intervention (although no studies have specifically reported on this) [82, 83]. Overcoming these and potentially providing a healthcare code incentive may help to advance adoption alongside additional evidence. Although it would seem that protocolisation would aid interpretation standardisation, early explorations have not proven this [84]. Computational quantification of the ICGFA signal [85–87] could also help, although this too has not yet been validated in clinical practice.

This meta-analysis has limitations. All included studies were English language only and the majority were retrospective in nature, with 2911 of the total 14,333 patients coming from RCTs. The potential advantages and disadvantages of including non-randomised studies in such an analysis have been debated extensively [88], but we felt it is important to present the totality of the evidence currently available to us as we eagerly await the publication of the two aforementioned recently concluded RCTs [26, 27]. This study also reports on the estimate effect size of ICGFA within RCTs only and in the non-randomised studies only, which both show a benefit. The risk of bias in non-RCTs was moderate for most and serious for a few, especially regarding confounding factors and patient selection, with many control groups coming from different time-periods, with our Harbord’s score test suggesting potential publication bias too. However, when sensitivity analysis was performed by removing the studies which were found to have a high risk of bias, there were no changes to the statistical significance of any findings. The predominance of patients underwent elective surgery for cancer so findings may not extrapolate to non-malignant or emergency surgeries. Studies too had variable intraoperative and post-operative practices and protocols, including ICGFA techniques and timing, as well as methods for assessing for AL with only small number of studies (*n* = 5) routinely assessing all patients with a contrast enema. These factors may not have been evenly divided between the groups in some of the non-RCTs; however, they were in the RCTs which perhaps explains some of the difference in statistical impact between RCTs and non-RCTs. The heterogeneity of reporting of these factors as well as the patient characteristics (e.g. age, BMI, comorbidities etc.) precluded any further subgroup analysis. The benefit of ICGFA may not only be limited to AL, but potentially other aspects such as anastomotic stricture rates; however, the primary focus of studies to date has been on AL.

## Conclusion

The results of this meta-analysis establish the current state of the clinical art regarding perfusion assessment with ICGFA and raise important aspects that the remaining RCTs may address. ICGFA is associated with significantly lower AL rates for colorectal resections overall and in rectal cancer resections, when used before and after anastomotic formation, and especially when agreeing with the surgeon’s initial assessment. If these findings are supported by upcoming RCT results, the use of ICGFA to differentiate lowest risk from higher risk patients can add rationale to its proposal for routine use in patients undergoing colorectal surgery.

## Supplementary Information

Below is the link to the electronic supplementary material.

Supplementary file1 (JPG 557 KB) Supplementary Figure 1. Traffic light plot for ROBINS-I.

Supplementary file2 (JPG 1015 KB) Supplementary Figure 2. Traffic light plot for ROB2.

Supplementary file3 (JPG 210 KB) Supplementary Figure 3. Forest plot showing odds ratio (OR) with 95% confidence interval (CI) of anastomotic leak in the ICGFA and non-ICGFA groups for studies where data was reported on the severity of AL.

Supplementary file4 (JPG 125 KB) Supplementary Figure 4. Forest plots showing odds ratio (OR) with 95% confidence interval (CI) regarding change of surgical strategy data for RCTs.

Supplementary file5 (JPG 49 KB) Supplementary Figure 5. Forest plots showing odds ratio (OR) with 95% confidence interval (CI) regarding change of mind data for RCTs reporting on rectal resections only.

Supplementary file6 (JPG 66 KB) Supplementary Figure 6. Extrapolated percentage of leaks expected in change of surgical plan group if no change in surgical strategy occurred (A) overall in all RCTs (B) rectal only RCTs.

Supplementary file7 (DOCX 43 KB) Supplementary Table 1. Study and patient demographics.

Supplementary file8 (DOCX 73 KB) Supplementary Table 2. Study, operative and post-operative characteristics.

## Data Availability

On request.
