# Supplementary figures and images for: Impact of indocyanine green fluorescence angiography on surgeon action and anastomotic leak in colorectal resections. A systematic review and meta-analysis
Source: Surg Endosc. 2025 Feb 3;39(3):1473–89. doi: 10.1007/s00464-025-11582-y (PMC11870979; doi:10.1007/s00464-025-11582-y)

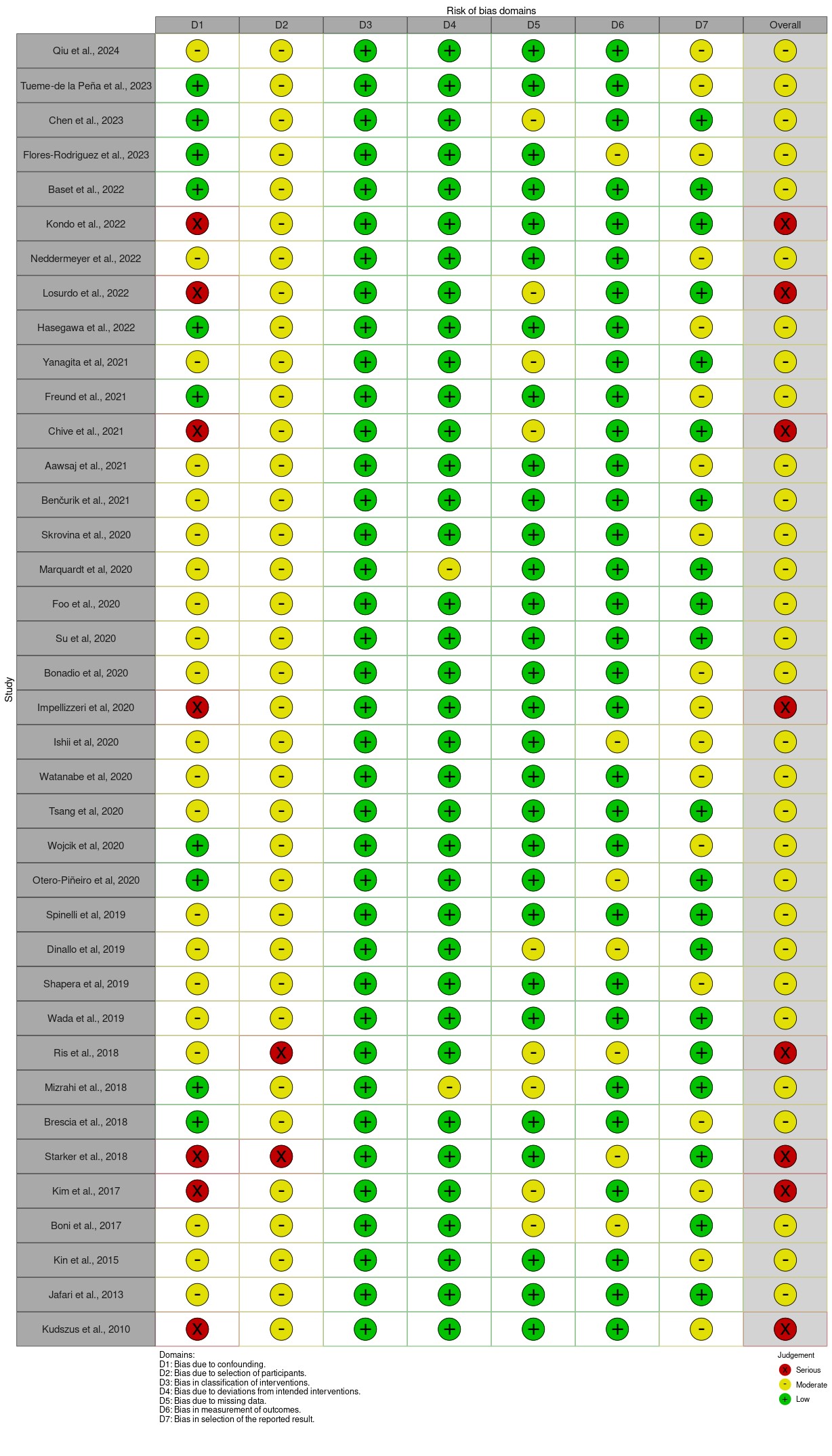

Supplement: Supplementary file 1 — Supplementary file1 (JPG 557 KB) Supplementary Figure 1. Traffic light plot for ROBINS-I. [file 464_2025_11582_MOESM1_ESM.jpg]

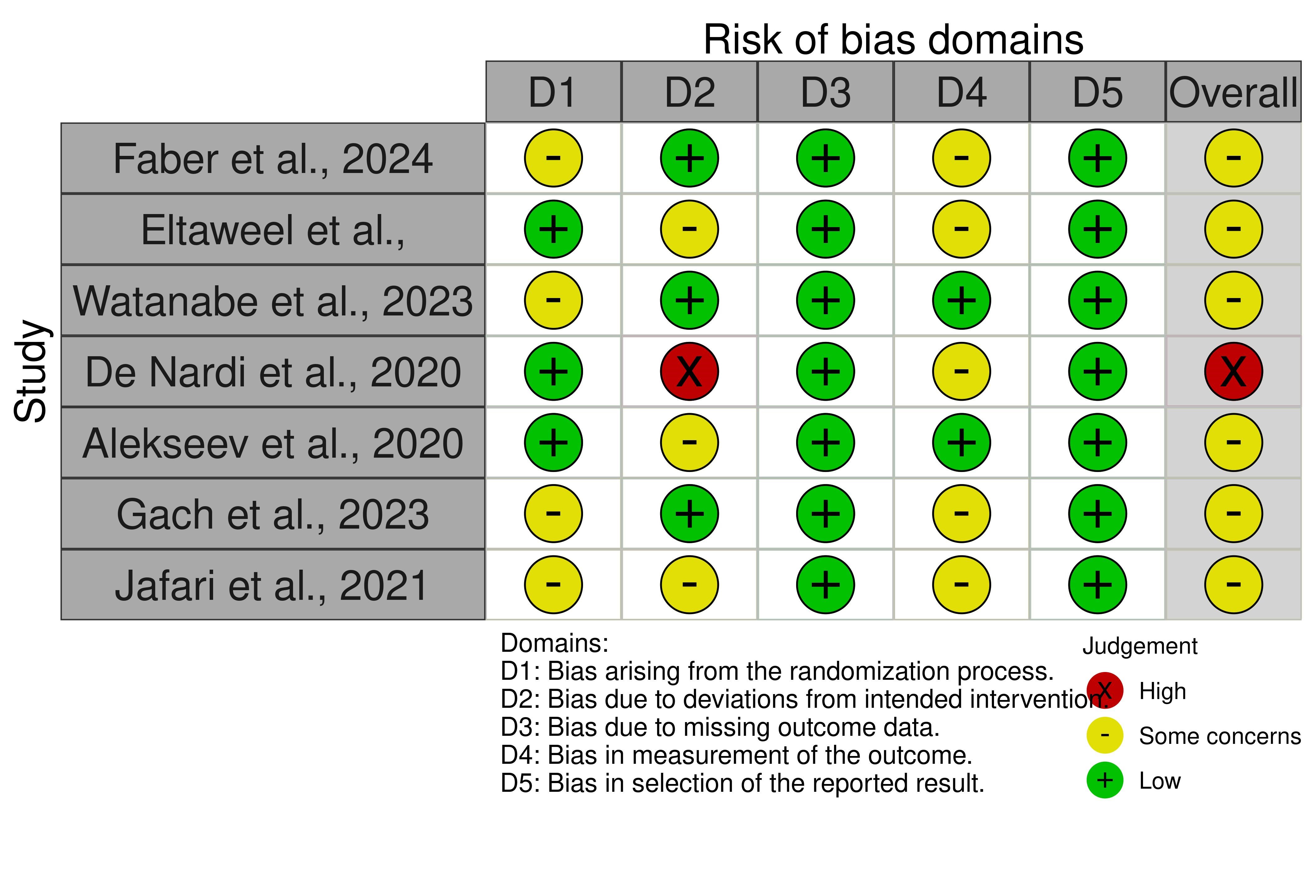

Supplement: Supplementary file 2 — Supplementary file2 (JPG 1015 KB) Supplementary Figure 2. Traffic light plot for ROB2. [file 464_2025_11582_MOESM2_ESM.jpg]

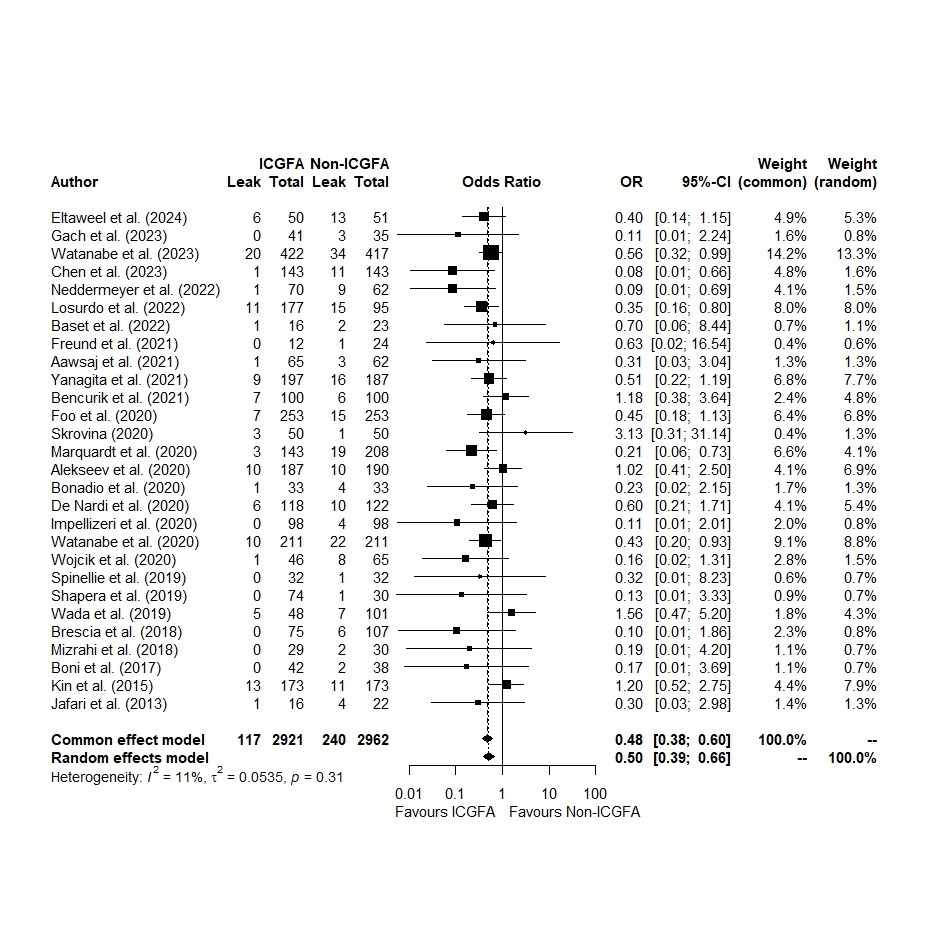

Supplement: Supplementary file 3 — Supplementary file3 (JPG 210 KB) Supplementary Figure 3. Forest plot showing odds ratio (OR) with 95% confidence interval (CI) of anastomotic leak in the ICGFA and non-ICGFA groups for studies where data was reported on the severity of AL. [file 464_2025_11582_MOESM3_ESM.jpg]

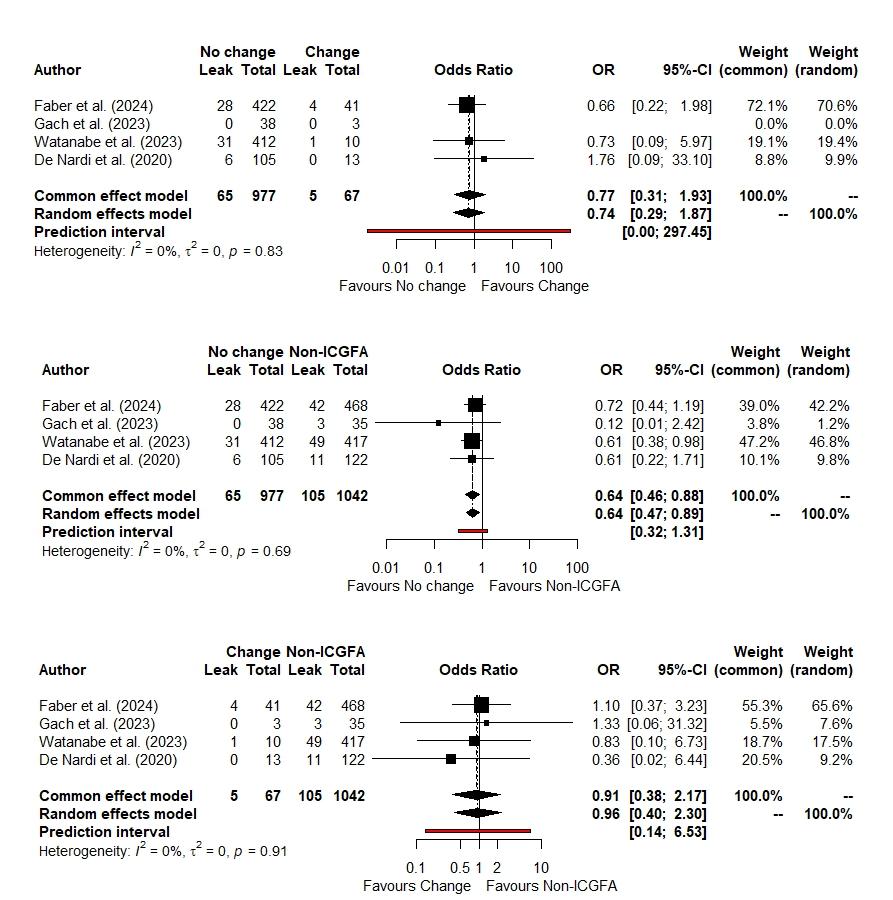

Supplement: Supplementary file 4 — Supplementary file4 (JPG 125 KB) Supplementary Figure 4. Forest plots showing odds ratio (OR) with 95% confidence interval (CI) regarding change of surgical strategy data for RCTs. [file 464_2025_11582_MOESM4_ESM.jpg]

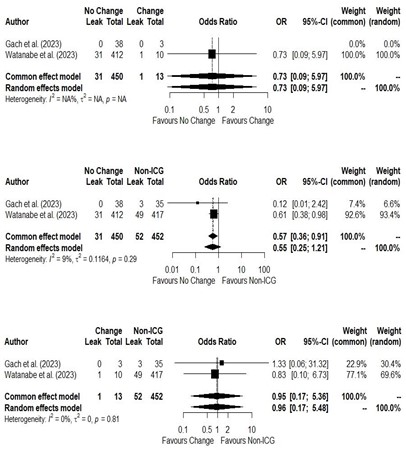

Supplement: Supplementary file 5 — Supplementary file5 (JPG 49 KB) Supplementary Figure 5. Forest plots showing odds ratio (OR) with 95% confidence interval (CI) regarding change of mind data for RCTs reporting on rectal resections only. [file 464_2025_11582_MOESM5_ESM.jpg]

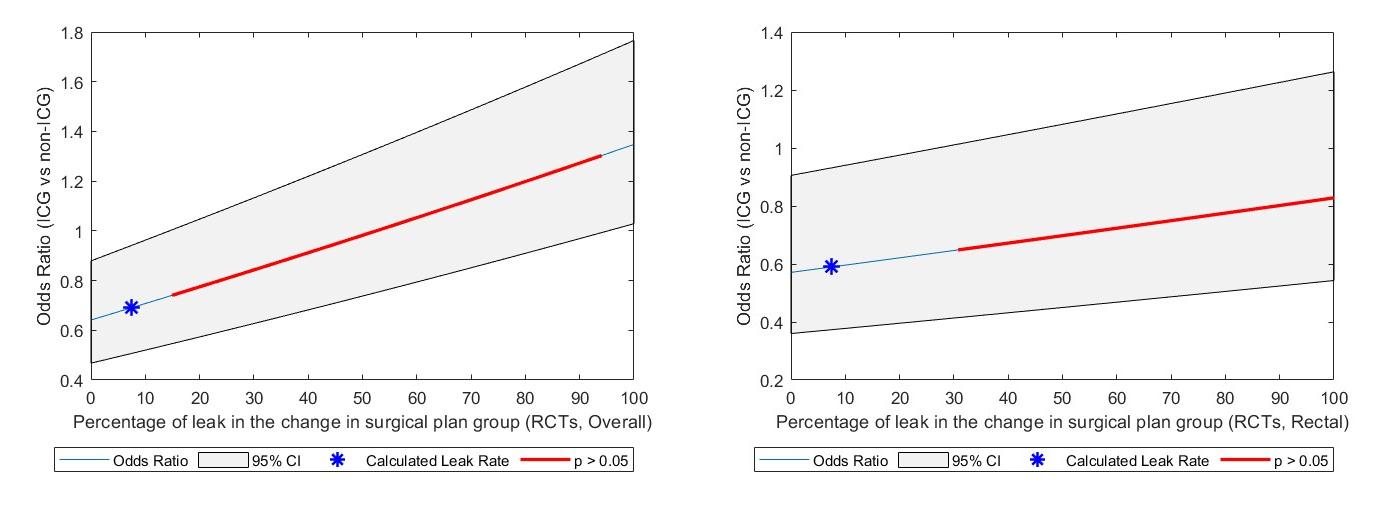

Supplement: Supplementary file 6 — Supplementary file6 (JPG 66 KB) Supplementary Figure 6. Extrapolated percentage of leaks expected in change of surgical plan group if no change in surgical strategy occurred (A) overall in all RCTs (B) rectal only RCTs. [file 464_2025_11582_MOESM6_ESM.jpg]
