# Supplementary material for: Impact of indocyanine green fluorescence angiography on surgeon action and anastomotic leak in colorectal resections. A systematic review and meta-analysis
Source: Surg Endosc. 2025 Feb 3;39(3):1473–89. doi: 10.1007/s00464-025-11582-y (PMC11870979; doi:10.1007/s00464-025-11582-y)
Supplement: Supplementary file 7 — Supplementary file7 (DOCX 43 KB) Supplementary Table 1. Study and patient demographics. [file 464_2025_11582_MOESM7_ESM.docx]

**Supplementary Table 1.** Study and patient demographics. BMI Body Mass Index, SD standard deviation, IQR interquartile range, CI confidence interval, NR not reported.

| **Study** | **Surgical site** | **Study group** | **N** | **Patient demographic characteristics** | | | | | | |
| --- | --- | --- | --- | --- | --- | --- | --- | --- | --- | --- |
|  |  |  |  | **Age (Years)** | **Sex (M:F)** | **BMI in kg/m^2^**  **(SD or Range)** | **Neoadjuvant Therapy (%)** | **Smoking (%)** | **Distance from anal verge (cm)** | **Diabetes (%)** |
| Faber et al., 2024 | Multiple sites throughout colon and rectum | ICGA | 463 | 69.0 (59-75 IQR) | 249:214 | 25.8 (23.5-29.2 IQR) | 5 | 10.6 | NR | 13 |
|  |  | Control | 468 | 67.5 (59-76 IQR) | 236:232 | 25.9 (23.4-29.0 IQR) | 5.3 | 12.6 | NR | 13.2 |
| Eltaweel et al., 2024 | Left including rectal | ICGA | 50 | 66.1 +/- 12.8 (SD) | 25:25 | 25.2 +/- 7.2 (SD) | 28 | NR | NR | 18 |
|  |  | Control | 51 | 65.1 +/- 11.3 (SD) | 28:23 | 25.6 +/- 8.7 (SD) | 23.5 | NR | NR | 21.6 |
| Qiu et al., 2024 | Rectal only | ICGA | 109 | 61 (55-68 IQR) | 42:67 | 24 (22.1-26 IQR) | 16.5 | 9.2 | 4 (3.5-4 IQR) | 14.7 |
|  |  | Control | 190 | 62 (55-68 IQR) | 74:116 | 23.7 (22-25.6 IQR) | 14.7 | 11.6 | 4 (3.5-4 IQR) | 16.8 |
| Gach et al., 2023 | Rectal only | ICGA | 41 | 64.7 +/- 10.5 (SD) | 26:15 | 26.5 +/- 47 (SD) | 70.7 | NR | 8.1 +/- 2.7 (SD) | NR |
|  |  | Control | 35 | 64.8 +/- 10.6 (SD) | 21:14 | 26.7 +/- 4.2 (SD) | 85.7 | NR | 7.6 +/- 2.4 (SD) | NR |
| Watanabe et al., 2023 | Rectal only | ICGA | 422 | 26/331 <75 | 266:156 | 313 < 25, 109 > 25 | 5.2 | NR | 72 (<5cm), 350 (>/=5cm) | NR |
|  |  | Control | 417 | 35/319 <75 | 274:143 | 312 < 25, 105 > 25 | 7.2 | NR | 52 (<5cm), 365 (>/=5cm) | NR |
| Tueme-de la Peña et al., 2023 | Multiple sites throughout colon and rectum | ICGA | 83 | 58 (49-65 SD) | 44:39 | 27 (25-29.7 SD) | 7.2 | 12 | NR | 4.8 |
|  |  | Control | 85 | 61 (52.5-69.5 SD) | 41:44 | 26.6 (24.3-28.7 SD) | 10.6 | 10.6 | NR | 12.9 |
| Chen et al., 2023 | Rectal only | ICGA | 143 | 69 (41-90 Range) | 73:70 | 23.9 (17.0-30.8 Range) | 55.9 | 9.1 | 3.8 (1.6-8.4 Range) | 7 |
|  |  | Control | 143 | 67 (40-88 Range) | 71:72 | 24.3 (17.4-30.8 Range) | 52.4 | 12.6 | 3.9 (2.0-8.3 Range) | 10.5 |
| Kondo et al., 2022 | Rectal only | ICGA | 73 | 67 (57-72 IQR) | 46:27 | 22.7 (20.7-25.1 IQR) | 11 | NR | 35 Upper, 8 Middle, 30 Lower | 24.7 |
|  |  | Control | 114 | 68 (59-73 IQR) | 77:37 | 22.2 (19.9-24.8 IQR) | 2.6 | NR | 47 Upper, 19 Middle, 48 Lower | 14.9 |
| Neddermeyer et al., 2022 | Left including rectal | ICGA | 70 | 66.5 (34-88 Range) | 48:22 | 26.7 (19.6-36.1 Range) | 24.3 | 13.4 | NR | 11.4 |
|  |  | Control | 62 | 59.5 (33-93 Range) | 39:23 | 26.7 (14.9-44.8 Range) | 24.2 | 21.7 | NR | 17.7 |
| Losurdo et al., 2022 | Left including rectal | ICGA | 177 | 69.9 +/- 11.2 (SD) | 109:68 | 25.6 +/- 4.3 (SD) | 38.4 | NR | NR | 20.3 |
|  |  | Control | 95 | 67.9 +/- 10.0 (SD) | 37:58 | 27.3 +/- 3.5 (SD) | 40 | NR | NR | 10.5 |
| Hasegawa et al., 2022 | Rectal only | ICGA | 66 | 60 (50-68 IQR) | 50:16 | 22.8 (21.1-25.6 IQR) | 25.8 | 68.2 | 5 (5.0-5.0 IQR) | NR |
|  |  | Control | 103 | 60 (51-68 IQR) | 81:22 | 23.2 (21.0-25.1 IQR) | 24.3 | 72.8 | 5 (4.5-5.5 IQR) | NR |
| Freund et al., 2021 | Redo ileocolic | ICGA | 12 | 53.5 (23-77 Range) | 5:7 | 24.1 (Median) | NR | 0 | NR | NR |
|  |  | Control | 24 | 58 (32-78 Range) | 13:11 | 23.6 (Median) | NR | 12.5 | NR | NR |
| Chive et al., 2021 | Multiple sites throughout colon and rectum | ICGA | 158 | 64 +/- 15 (SD) | 95:63 | 26.1 +/- 4.5 (SD) | NR | NR | NR | NR |
|  |  | Control | 677 | 62 +/- 16 (SD) | 374:303 | 26.5 +/- 6.5 (SD) | NR | NR | NR | NR |
| Aawsaj et al., 2021 | Right only | ICGA | 65 | 69.5 +/- 7.9 (SD) | 22:43 | 27.6 +/- 8.2 (SD) | NR | NR | NR | NR |
|  |  | Control | 62 | 68.9 +/- 9.1 (SD) | 32:30 | 28.3 +/- 8.5 (SD) | NR | NR | NR | NR |
| Jafari et al., 2021 | Rectal only | ICGA | 178 | 57.2 +/- 11.4 (SD) | 109:69 | 27.8 +/- 5.6 (SD) | 63.5 | 27.5 | 27 Upper, 145 Middle/Lower | 12.4 |
|  |  | Control | 169 | 57.0 +/- 11.4 (SD) | 99:70 | 28.2 +/- 5.9 (SD) | 65.7 | 18.3 | 24 Upper, 143 Middle/Lower | 13.6 |
| Kudszus et al., 2010 | Multiple sites throughout colon and rectum | ICGA | 201 | 67.8 +/- 25.2 (Range) | 85:116 | 25.3 +/- 8.4 (Range) | NR | NR | NR | NR |
|  |  | Control | 201 | 69.0 +/- 21.8 (Range) | 85:116 | 25.7 +/- 7.8 (Range) | NR | NR | NR | NR |
| Yanagita et al, 2021 | Left including rectal | ICGA | 197 | 70 (34-93 Range) | 116:81 | 22.3 (13.2-31.9 Range) | 3.6 | 16.8 | NR | NR |
|  |  | Control | 187 | 69 (38-94 Range) | 115:72 | 22.0 (15.2-34.5 Range) | 1.6 | 21.4 | NR | NR |
| Benčurik et al., 2021 | Rectal only | ICGA | 100 | 62.6 +/- 9.7 (SD) | 66:34 | 26.8 +/- 4.5 (SD) | 66 | 25 | 7.1 +/- 2.0 (SD) | 18 |
|  |  | Control | 100 | 64.4 +/- 9.2 (SD) | 64:36 | 27.3 +/- 4.6 (SD) | 77 | 12 | 7.1 +/- 2.4 (SD) | 18 |
| Skrovina et al., 2020 | Rectal only | ICGA | 50 | 62.4 +/- 9.0 (SD) | 34:16 | 27 +/-4 (SD) | 68 | 28 | 4.6 +/- 1.3 (SD) | 16 |
|  |  | Control | 50 | 65.0 +/- 9.4 (SD) | 29:21 | 27 +/- 5 (SD) | 74 | 10 | 4.6 +/- 1.5 (SD) | 20 |
| Marquardt et al, 2020 | Right and rectal | ICGA | 143 | 69 (58-76 IQR) (Rectal), 74 (65-80 IQR) (Right) | 35:32 (Rectal), 31:45 (Right) | 25.2 (23-27.6 IQR) (Rectal), 25.8 (21.7-29.3 IQR) (Right) | 21.7 | NR | NR | NR |
|  |  | Control | 208 | 71 (60-78 IQR) (Rectal), 77 (69-82 IQR) (Right) | 37:32 (Rectal), 75:74 (Right) | 26.5 (23.9-31.1 IQR) (Rectal), 26 (23-29.6 IQR) (Right) | 15.4 | NR | NR | NR |
| Su et al, 2020 | Multiple sites throughout colon and rectum | ICGA | 84 | 59.1 +/- 11.1 (SD) | 48:36 | 24.6 +/- 3.4 (SD) | 16.7 | NR | NR | NR |
|  |  | Control | 105 | 60.2 +/- 9.8 (SD) | 55:50 | 23.8 +/- 2.7 (SD) | 28.6 | NR | NR | NR |
| Alekseev et al, 2020 | Left including rectal | ICGA | 187 | 63 (21-86 Range) | 92:95 | NR | 10.7 | NR | NR | 8 |
|  |  | Control | 190 | 63 (66-85 Range) | 92:98 | NR | 10 | NR | NR | 8.9 |
| Bonadio et al, 2020 | Rectal only | ICGA | 33 | 71.85 +/- 11.1 (SD) | 21:12 | 25.6 +/- 4.0 (SD) | 48.5 | NR | NR | 18.2 |
|  |  | Control | 33 | 69.03 +/- 11.3 (SD) | 15:18 | 25.7 +/- 4.1 (SD) | 42.4 | NR | NR | 6.1 |
| De Nardi et al, 2020 | Left including rectal | ICGA | 118 | 66.1 (Mean) | 60:58 | 25.6 (Mean) | 23.7 | NR | NR | 14.4 |
|  |  | Control | 122 | 65.1 (Mean) | 66:56 | 25.2 (Mean) | 17.2 | NR | NR | 15.6 |
| Impellizzeri et al, 2020 | Left including rectal | ICGA | 98 | 66 (59-74 IQR) | 53:45 | 47 (</= 25), 41 (26-30), 10 (>30) | 13.3 | NR | NR | 9.2 |
|  |  | Control | 98 | 71 (58-79 IQR) | 57:41 | 88 (</= 25), 7 (26-30), 3 (>30) | 11.2 | NR | NR | 7.1 |
| Ishii et al, 2020 | Multiple sites throughout colon and rectum | ICGA | 223 | 67 (30-90 Range) | 126:97 | 22.9 (15.1-32.3 Range) | 17.5 | NR | 4.0 (2-12 Range) | NR |
|  |  | Control | 265 | 69 (27-93 Range) | 136:129 | 22.7 (16.1-33.4 Range) | 8.7 | NR | 4.5 (2-12 Range) | NR |
| Watanabe et al, 2020 | Rectal only | ICGA | 211 | 66 (34-92 Range) | 128:83 | 22.3 (15.6-36.4 Range) | 19.9 | NR | 10.0 (2.5-15 Range) | 22.3 |
|  |  | Control | 211 | 66 (36-89 Range) | 131:80 | 22.4 (14.5-31.6 Range) | 23.2 | NR | 10.0 (3-15 Range) | 23.2 |
| Tsang et al, 2020 | Multiple sites throughout colon and rectum | ICGA | 62 | 69.82 +/- 9.89 (SD) | 39:23 | 23.47 +/- 3.81 (SD) | 6.5 | 14.5 | NR | 38.7 |
|  |  | Control | 69 | 67.71 +/- 11.65 (SD) | 47:22 | 22.36 +/- 3.62 (SD) | 5.8 | 10.1 | NR | 24.6 |
| Wojcik et al, 2020 | Left including rectal | ICGA | 46 | 65.7 +/- 11.1 (SD) | 30:16 | 25.8 +/- 3.4 (SD) | 41.3 | 10.9 | NR | 6.5 |
|  |  | Control | 65 | 68.6 +/- 12 (SD) | 40:25 | 26.2 +/- 4.6 (SD) | 46.2 | 20 | NR | 9.2 |
| Otero-Piñeiro et al, 2020 | Rectal only | ICGA | 80 | 68.0 +/0 11.4 (SD) | 51:29 | 26.1 +/- 4.1 (SD) | 46.3 | 25 | 8.79 +/- 3.3 (SD) | 21.3 |
|  |  | Control | 204 | 66.6 +/- 12.3 (SD) | 123:81 | 25.4 +/- 3.9 (SD) | 55.4 | 30.9 | 8.18 +/- 3.4 (SD) | 13.7 |
| Spinelli et al, 2019 | IPAA only | ICGA | 32 | 39.41 +/- 14.09 (SD) | 21:11 | 22.16 +/- 0.7 (SD) | NR | NR | NR | NR |
|  |  | Control | 32 | 45.75 +/- 15.9 (SD) | 17:15 | 22.84 +/- 0.52 (SD) | NR | NR | NR | NR |
| Dinallo et al, 2019 | Multiple sites throughout colon and rectum | ICGA | 234 | 61.5 (34.6-88.4 95% CI) | 108:126 | 28.3 (15.6-41.0 95% CI) | 6.8 | 15.8 | NR | 11.7 |
|  |  | Control | 320 | 62.5 (35.3-89.7 95% CI) | 138:182 | 28.3 (10-46.6 95% CI) | 7.5 | 15.9 | NR | 18.1 |
| Shapera et al, 2019 | Left including rectal | ICGA | 74 | 58 (Mean) | 42:32 | 27 (Mean) | 10.8 | NR | NR | 10.8 |
|  |  | Control | 30 | 60 (Mean) | 17:13 | 28 (Mean) | 30 | NR | NR | 13.3 |
| Boni et al., 2017 | Rectal only | ICGA | 42 | 69 +/- 8 (SD) | 28:14 | 27 +/- 11 (SD) | 78.6 | 42.9 | 6.3 +/- 2 (SD) | NR |
|  |  | Control | 38 | 67 +/- 7 (SD) | 22:16 | 29 +/- 15 (SD) | 60.5 | 60.5 | 7.2 +/- 3 (SD) | NR |
| Kin et al., 2015 | Left including rectal | ICGA | 173 | 58.2 +/- 13.2 (SD) | 93:80 | 27.0 +/- 4.9 (SD) | 20.2 | 9.8 | NR | 12.7 |
|  |  | Control | 173 | 58.1 +/- 13.2 (SD) | 93:80 | 26.5 +/- 5.3 (SD) | 20.2 | 11 | NR | 9.8 |
| Jafari et al., 2013 | Rectal only | ICGA | 16 | 58 (Mean) | 12:4 | 27 (Mean) | 62.5 | 12.5 | NR | 0 |
|  |  | Control | 22 | 63 (Mean) | 16:6 | 27 (Mean) | 68.2 | 27.3 | NR | 18.2 |
| Kim et al., 2017 | Rectal only | ICGA | 310 | 58 +/- 11 (SD) | 182:128 | 23.9 +/- 2.9 (SD) | 31 | NR | 5.4 +/- 3.3 (SD) | NR |
|  |  | Control | 347 | 57 +/- 11 (SD) | 216:131 | 23.9 +/- 3.7 (SD) | 30 | NR | 5.9 +/- 3 (SD) | NR |
| Wada et al., 2019 | Rectal only | ICGA | 48 | 66 (Median) | 31:17 | 22.5 (Median) | 10.4 | 31.3 | NR | 14.6 |
|  |  | Control | 101 | 67 (Median) | 70:31 | 21.4 (Median) | 21.8 | 28.7 | NR | 11.9 |
| Ris et al., 2018 | Multiple sites throughout colon and rectum | ICGA | 504 | 64 (18-88 Range) | 279:225 | 25 (13-57 Range) | NR | NR | NR | NR |
|  |  | Control | 1173 | NR | NR | NR | NR | NR | NR | NR |
| Mizrahi et al., 2018 | Rectal only | ICGA | 29 | 58 +/- 12 (SD) | 16:14 | 25.9 +/- 5.7 (SD) | 58.6 | 6.7 | 2.7 +/- 1.1 (SD) | 6.7 |
|  |  | Control | 30 | 58 +/- 13 (SD) | 18:12 | 27.2 +/- 6.2 (SD) | 46.7 | 0 | 2.9 +/- 1.2 (SD) | 10 |
| Foo et al., 2020 | Left including rectal | ICGA | 253 | 66.6 +/- 10.6 (SD) | 166:87 | NR | 19.4 | 15.4 | 12.6 +/- 7.9 (SD) | NR |
|  |  | Control | 253 | 67.2 +/- 11.0 (SD) | 163:90 | NR | 23.7 | 7.1 | 17.8 +/- 44.2 (SD) | NR |
| Flores-Rodriguez et al., 2023 | Multiple sites throughout colon and rectum | ICGA | 280 | 70.5 (Mean) | 171:109 | NR | 6.4 | NR | NR | 19.6 |
|  |  | Control | 505 | 71.7 (Mean) | 299:206 | NR | 4.4 | NR | NR | 17 |
| Baset et al., 2022 | Multiple sites throughout colon and rectum | ICGA | 16 | 49.3 +/- 8.1 (SD) | 7:9 | 28.6 +/- 1.5 (SD) | 50 | NR | NR | NR |
|  |  | Control | 23 | 51.1 +/- 10.4 (SD) | 11:12 | 27.7 +/- 1.7 (SD) | 43.5 | NR | NR | NR |
| Brescia et al., 2018 | Multiple sites throughout colon and rectum | ICGA | 75 | 67.1 +/- 6 (SD) | 43:32 | 24.4 +/-3 (SD) | NR | 20 | NR | NR |
|  |  | Control | 107 | 65.7 +/- 7 (SD) | 63:44 | 25.6 +/- 3 (SD) | NR | 23.4 | NR | NR |
| Starker et al., 2017 | Multiple sites throughout colon and rectum | ICGA | 238 | 62.4 (Mean) | 126:112 | 28.4 (Mean) | NR | 16.8 | NR | 2.5 |
|  |  | Control | 109 | 60.8 (Mean) | 50:59 | 27.4 (Mean) | NR | 14.7 | NR | 5.5 |
