# Supplementary material for: Impact of indocyanine green fluorescence angiography on surgeon action and anastomotic leak in colorectal resections. A systematic review and meta-analysis
Source: Surg Endosc. 2025 Feb 3;39(3):1473–89. doi: 10.1007/s00464-025-11582-y (PMC11870979; doi:10.1007/s00464-025-11582-y)
Supplement: Supplementary file 8 — Supplementary file8 (DOCX 73 KB) Supplementary Table 2. Study, operative and post-operative characteristics. [file 464_2025_11582_MOESM8_ESM.docx]

**Supplementary Table 2.** Study, operative and post-operative characteristics. NR - not reported, CS - certified surgeon, EP - educational procedure, IC - ileocaecal resection, RC - right hemicolectomy, TC - transverse colectomy, LC - left hemicolectomy, SC - sigmoid colectomy, AR - anterior resection, HAR - high anterior resection, LAR - low anterior resection, ULAR - ultra-low anterior resection, ISR - intersphincteric resection, TME - total mesorectal excision, Ta - transanal total mesorectal excision, Lap - laparoscopic, Rob - robotic, O - open, Con - conversion, A/B/C - International Study Group of Rectal Cancer grading of anastomotic leakage, CD - Clavien-Dindo classification, NA - not applicable.

| **Study** | **Surgical site** | **Elective/ Emergency** | **ICG Use** | **Mechanical bowel preparation** | **Study group** | **N** | **Operative characteristics** | | | | | **Post-operative characteristics** | | | |
| --- | --- | --- | --- | --- | --- | --- | --- | --- | --- | --- | --- | --- | --- | --- | --- |
|  |  |  |  |  |  |  | **Surgeon Experience (n)** | **Method of Surgical Access (%)** | **Operation Performed (%)** | **Splenic flexure takedown (%)** | **Diverting stoma (%)** | **AL assessment method** | **AL rate**  **(n (%))** | **AL severity (n)** | **Anastomotic stricture**  **(n (%))** |
| Faber et al., 2024 | Multiple sites throughout colon and rectum | Elective only | Before and after | NR | ICGA | 463 | NR | Lap/Rob/Con  78/22/7 | IC/RC/TC/LC/SC/LAR/Other 2/38/1/9/29/19/2 | NR | NR | Clinical | 32 (6.9) | NR | NR |
|  |  |  |  |  | Control | 468 |  | Lap/Rob/Con  78/22/7 | IC/RC/TC/LC/SC/LAR/Other  3/41/1/8/26/21/0 |  |  |  | 42 (9) |  |  |
| Eltaweel et al., 2024 | Left including rectal | Elective only | Before and after | Yes, with oral antibiotics | ICGA | 50 | NR | NR | LC/LAR  52/48 | 100 | NR | Clinical | 7 (14) | A/B/C  1/2/4 | 1 (2) |
|  |  |  |  |  | Control | 51 |  |  | LC/LAR  56.9/43.1 | 100 |  |  | 16 (31.4) | A/B/C  3/5/8 | 3 (5.9) |
| Qiu et al., 2024 | Rectal only | Elective only | Before and after | NR | ICGA | 109 | > 100 cases of Lap ISR | Lap  100 | ISR  100 | NR | 81.6 | Contrast enema for all cases | 4 (3.7) | A+B/C  4/0 | 1 (0.9) |
|  |  |  |  |  | Control | 190 |  | Lap  100 | ISR  100 |  | 81.6 |  | 20 (10.5) | A+B/C  15/5 | 4 (2.1) |
| Gach et al., 2023 | Rectal only | Elective only | Before and after | Yes | ICGA | 41 | NR | Lap  100 | AR  100 | NR | 0 | Clinical | 0 (0) | A/B/C  0/0/0 | NR |
|  |  |  |  |  | Control | 35 |  | Lap  100 | AR  100 |  | 0 |  | 3 (8.6) | A/B/C  0/1/2 |  |
| Watanabe et al., 2023 | Rectal only | Elective only | Before only | NR | ICGA | 422 | >30 cases of laparoscopic rectal cancer surgery | Lap/Rob/Ta/Con 43.6/47.9/8.5/0.7 | HAR/LAR/ISR 12.1/73/14.9 | NR | 50.4 | Clinical if no stoma, contrast enema for all stoma cases | 32 (7.6) | A/B+C  12/20 | NR |
|  |  |  |  |  | Control | 417 |  | Lap/Rob/Ta/Con 45.6/44.1/10.3/0.7 | HAR/LAR/ISR 9.8/75.3/14.9 |  | 54.9 |  | 49 (11.8) | A/B+C  15/34 |  |
| Tueme-de la Peña et al., 2023 | Multiple sites throughout colon and rectum | Elective and emergency | Before and after | NR | ICGA | 83 | NR | Lap  100 | RC/LC/SC/LAR/ULAR  16.9/15.7/37.3/24.1/6 | NR | 16.9 | Clinical | 5 (6) | NR | NR |
|  |  |  |  |  | Control | 85 |  | Lap  100 | RC/LC/SC/LAR/ULAR  32.9/9.4/36.5/5.9/15.3 |  | 20 |  | 6 (7.1) |  |  |
| Chen et al., 2023 | Rectal only | Elective only | Before and after | NR | ICGA | 143 | NR | Lap  100 | Ta  100 | NR | 85.3 | Contrast enema within 30 days | 5 (3.5) | A/B/C  4/1/0 | NR |
|  |  |  |  |  | Control | 143 |  | Lap  100 | Ta  100 |  | 86.7 |  | 23 (16.1) | A/B/C  12/10/1 |  |
| Kondo et al., 2022 | Rectal only | Elective only | Before only | NR | ICGA | 73 | NR | Lap  100 | HAR/LAR/ISR  24.7/61.7/13.7 | NR | 42.5 | Clinical | 3 (4.1) | NR | NR |
|  |  |  |  |  | Control | 114 |  | Lap  100 | HAR/LAR/ISR  28.9/64/7 |  | 35.1 |  | 14 (12.3) |  |  |
| Neddermeyer et al., 2022 | Left including rectal | Elective only | Before and after | Yes, with oral antibiotics | ICGA | 70 | CS/EP  48/22 | Lap or Rob/O  56/14 | SC/TME  54.3/45.7 | NR | 45.7 | Clinical | 1 (1.4) | A/B/C  0/0/1 | NR |
|  |  |  |  |  | Control | 62 | CS/EP  43/19 | Lap or Rob/O  42/20 | SC/TME  51.6/48.4 |  | 46.8 |  | 9 (14.5) | A/B/C  0/1/8 |  |
| Losurdo et al., 2022 | Left including rectal | Elective only | Before and after | Yes, for rectal lesions | ICGA | 177 | Senior surgeon or supervised trainee | Lap  100 | NR | 100 | 44.1 | Clinical if no stoma, contrast enema for all stoma cases | 19 (10.8) | A/B/C  8/6/5 | NR |
|  |  |  |  |  | Control | 95 |  | Lap  100 |  | 100 | 32.6 |  | 17 (17.8) | A/B/C  2/10/5 |  |
| Hasegawa et al., 2022 | Rectal only | Elective only | Before only | NR | ICGA | 66 | >200 rectal cancer surgeries | Lap  100 | ISR  100 | NR | 100 | Clinical | 3 (4.5) | NR | 0 (0) |
|  |  |  |  |  | Control | 103 |  | Lap  100 | ISR  100 |  | 97.1 |  | 20 (19.4) |  | 4 (3.9) |
| Freund et al., 2021 | Redo ileocolic | Elective only | Before and after | NR | ICGA | 12 | NR | Lap/O/Con  91.7/8.3/18.1 | Redo Ileocolic  100 | NA | NR | Clinical | 0 (0) | A/B/C  0./0/0 | NR |
|  |  |  |  |  | Control | 24 |  | Lap/O/Con  95.8/4.2/30.4 | Redo ileocolic  100 |  |  |  | 1 (4.2) | A/B/C  0/0/1 |  |
| Chive et al., 2021 | Multiple sites throughout colon and rectum | Elective only | Before only | NR | ICGA | 158 | NR | Lap/O  70.9/29.1 | NR | NR | NR | Clinical | 3 (1.9) | NR | NR |
|  |  |  |  |  | Control | 677 |  | Lap/O  46.2/53.8 |  |  |  |  | 39 (5.8) |  |  |
| Aawsaj et al., 2021 | Right only | NR | Before and after | NR | ICGA | 65 | NR | Lap/O/Con  81.5/18.5/11.3 | RC  100 | NA | 0 | Clinical | 1 (1.5) | A/B/C  0/0/1 | NR |
|  |  |  |  |  | Control | 62 |  | Lap/O/Con  90.3/9.7/8.9 | RC  100 |  | 0 |  | 3 (4.8) | A/B/C  0/1/2 |  |
| Jafari et al., 2021 | Rectal only | Elective only | Before and after | NR | ICGA | 178 | NR | Lap/Rob/O/Con  47.8/36.5/15.7/8.7 | LAR  100 | 75.8 | 73.6 | Clinical | 16 (9) | NR | NR |
|  |  |  |  |  | Control | 169 |  | Lap/Rob/O/Con  45/41.4/13.6/5.5 | LAR  100 | 82.8 | 80.5 |  | 16 (9.5) |  |  |
| Kudszus et al., 2010 | Multiple sites throughout colon and rectum | Elective and emergency | Before and after | NR | ICGA | 201 | 10.2 Years | NR | NR | NR | NR | Clinical | 7 (3.5) | NR | NR |
|  |  |  |  |  | Control | 201 | 11.4 Years |  |  |  |  |  | 15 (7.5) |  |  |
| Yanagita et al, 2021 | Left including rectal | Elective only | Before only | NR | ICGA | 197 | NR | Lap/Rob/O/Con  93.9/4.1/2/1 | LC/SC/HAR/LAR  3.6/33/31/32.5 | NR | 22.8 | Clinical | 9 (4.6) | B/C  7/2 | NR |
|  |  |  |  |  | Control | 187 |  | Lap/Rob/O/Con  80.7/1.1/18.2/2 | LC/SC/HAR/LAR  10.7/33.7/31.6/24.1 |  | 11.8 |  | 16 (8.6) | B/C  6/10 |  |
| Benčurik et al., 2021 | Rectal only | NR | Before only | NR | ICGA | 100 | NR | Lap/Rob  58/42 | LAR  100 | NR | 100 | Contrast enema for all cases | 9 (9) | A/B/C  2/4/3 | NR |
|  |  |  |  |  | Control | 100 |  | Lap/Rob  72/28 | LAR  100 |  | 100 |  | 19 (19) | A/B/C  13/1/5 |  |
| Skrovina et al., 2020 | Rectal only | Elective only | Before only | NR | ICGA | 50 | NR | Lap/Rob  35/15 | LAR  100 | NR | 100 | Contrast enema within 30 days | 5 (10) | A/B/C  2/2/1 | NR |
|  |  |  |  |  | Control | 50 |  | Lap/Rob  37/13 | LAR  100 |  | 100 |  | 9 (18) | A/B/C  8/1/0 |  |
| Marquardt et al, 2020 | Right and rectal | Elective and emergency | Before and after | Yes, with oral antibiotics | ICGA | 143 | NR | NR | RC/LAR  76/67 | NR | 34.3 | Clinical | 4 (2.8) | A/B/C  1/0/3 | NR |
|  |  |  |  |  | Control | 208 |  | NR | RC/LAR  149/59 |  | 26.7 |  | 20 (9.6) | A/B/C  1/1/18 |  |
| Su et al, 2020 | Multiple sites throughout colon and rectum | NR | Before and after | NR | ICGA | 84 | NR | Lap  100 | RC/TC/LC  65.5/11.9/22.6 | NR | NR | Clinical | 0 (0) | NA | 0 (0) |
|  |  |  |  |  | Control | 105 |  | Lap  100 | RC/TC/LC  68.6/8.6/22.9 |  |  |  | 0 (0) | NA | 0 (0) |
| Alekseev et al, 2020 | Left including rectal | Elective only | Before only | NR | ICGA | 187 | >50 rectal resections per year | Lap/O  46.5/53.5 | LC/HAR/LAR  9.1/31.6/59.4 | 27.3 | 71.1 | Contrast enema within 30 days | 17 (9.1) | A/B/C  7/6/4 | NR |
|  |  |  |  |  | Control | 190 |  | Lap/O  40.5/59.5 | LC/HAR/LAR  13.2/31.6/55.3 | 25.3 | 70.5 |  | 31 (16.3) | A/B/C  21/7/3 |  |
| Bonadio et al, 2020 | Rectal only | Elective only | Before and after | NR | ICGA | 33 | NR | Lap  100 | AR  100 | NR | 57.6 | Clinical if no stoma, contrast enema for all stoma cases | 2 (6.1) | A/B/C  1/1/0 | NR |
|  |  |  |  |  | Control | 33 |  | Lap  100 | AR  100 |  | 42.4 |  | 7 (21.2) | A/B/C  3/1/3 |  |
| De Nardi et al, 2020 | Left including rectal | Elective and emergency | Before and after | Yes, for LAR | ICGA | 118 | NR | Lap  100 | LC/LAR  52.5/47.5 | 100 | 21.2 | Clinical | 6 (5.1) | A/B/C  0/2/4 | 0 (0) |
|  |  |  |  |  | Control | 122 |  | Lap  100 | LC/LAR  56.6/43.4 | 100 | 20.5 |  | 11 (9) | A/B/C  1/3/7 | 1 (0.8) |
| Impellizzeri et al, 2020 | Left including rectal | Elective only | Before and after | NR | ICGA | 98 | NR | Lap  100 | LC/SC/LAR  31.6/30.6/37.8 | NR | 30.6 | Clinical | 0 (0) | A/B/C  0/0/0 | 0 (0) |
|  |  |  |  |  | Control | 98 |  | Lap  100 | LC/SC/LAR  19.4/43.9/36.7 |  | 32.7 |  | 6 (6.1) | A/B/C  2/0/4 | 1 (1) |
| Ishii et al, 2020 | Multiple sites throughout colon and rectum | NR | Before only | NR | ICGA | 223 | NR | NR | NR | NR | NR | Clinical | 4 (1.8) | NR | NR |
|  |  |  |  |  | Control | 265 |  |  |  |  |  |  | 14 (5.3) |  |  |
| Watanabe et al, 2020 | Rectal only | Elective only | Before only | No | ICGA | 211 | NR | Lap  100 | LAR  100 | NR | 50.7 | Clinical | 10 (4.7) | CD II/CD III  4/6 | NR |
|  |  |  |  |  | Control | 211 |  | Lap  100 | LAR  100 |  | 52.1 |  | 22 (10.4) | CD II/CD III  2/20 |  |
| Tsang et al, 2020 | Multiple sites throughout colon and rectum | Elective only | Before only | Yes, with oral antibiotics | ICGA | 62 | NR | Lap/Rob/O  3.2/93.6/3.2 | RC/LC/HAR/LAR  33.9/6.5/30.6/29 | NR | NR | Clinical | 2 (3.2) | NR | NR |
|  |  |  |  |  | Control | 69 |  | Lap/Rob/O  72.5/17.4/10.1 | RC/LC/HAR/LAR  33.3/13/30.4/23.2 |  |  |  | 3 (4.3) |  |  |
| Wojcik et al, 2020 | Left including rectal | Elective only | Before only | Yes, for rectal resection | ICGA | 46 | NR | Lap/O/Con  95.7/4.3/9.1 | NR | 100 | 52.2 | Clinical | 3 (6.5) | A/B+C  2/1 | NR |
|  |  |  |  |  | Control | 65 |  | Lap/O/Con  89.2/10.8/1.7 |  | 100 | 60 |  | 11 (16.9) | A/B+C  3/8 |  |
| Otero-Piñeiro et al, 2020 | Rectal only | Elective only | Before and after | NR | ICGA | 80 | NR | Lap  100 | HAR/LAR/ULAR  22.5/65/12.5 | 33.8 | 72.5 | Clinical | 2 (2.5) | NR | NR |
|  |  |  |  |  | Control | 204 |  | Lap  100 | HAR/LAR/ULAR  26/55.4/18.6 | 34.3 | 72.1 |  | 23 (11.3) |  |  |
| Spinelli et al, 2019 | IPAA only | Elective only | Before and after | NR | ICGA | 32 | NR | Lap/O/Con  100/0/12.5 | Ileal pouch-anal anastomosis 100 | NA | 100 | Clinical | 0 (0) | A/B/C  0/0/0 | NR |
|  |  |  |  |  | Control | 32 |  | Lap/O/Con  50/50/12.5 | Ileal pouch-anal anastomosis 100 |  | 100 |  | 1 (3.1) | A/B/C  0/1/0 |  |
| Dinallo et al, 2019 | Multiple sites throughout colon and rectum | Elective and emergency | Before only | NR | ICGA | 234 | NR | Lap/Rob  92.7/7.3 | RC/TC/LC/LAR/Other  27.4/1.3/23.5/42.7/5.1 | 42.7 | 6 | Clinical | 3 (1.3) | NR | NR |
|  |  |  |  |  | Control | 320 |  | Lap/Rob  91.6/8.4 | RC/TC/LC/LAR/Other  26.3/0.9/31.6/34.1/7.2 | 34.1 | 6.3 |  | 4 (1.3) |  |  |
| Shapera et al, 2019 | Left including rectal | Elective only | Before and after | NR | ICGA | 74 | NR | Rob  100 | LC/SC/LAR  8.1/13.5/78.4 | NR | NR | Clinical | 0 (0) | A/B/C  0/0/0 | 0 (0) |
|  |  |  |  |  | Control | 30 |  | Rob  100 | LC/SC/LAR  3.3/20/76.7 |  |  |  | 1 (3.3) | A/B/C  0/0/1 | 1 (3.3) |
| Boni et al., 2017 | Rectal only | Elective only | Before only | NR | ICGA | 42 | NR | Lap  100 | LAR  100 | NR | 100 | Clinical | 0 (0) | A/B/C  0/0/0 | NR |
|  |  |  |  |  | Control | 38 |  | Lap  100 | LAR  100 |  | 100 |  | 2 (5) | A/B/C  0/1/1 |  |
| Kin et al., 2015 | Left including rectal | Elective only | Before only | At the discretion of the senior surgeon | ICGA | 173 | NR | Lap/O  64/36 | LC/SC/HAR/LAR/ULAR  6/40/34/17/3 | NR | 17 | Clinical | 13 (7.5) | B/C  6/7 | NR |
|  |  |  |  |  | Control | 173 |  | Lap/O  42/58 | LC/SC/HAR/LAR/ULAR  3/43/34/17/3 |  | 17 |  | 11 (6.4) | B/C  7/4 |  |
| Jafari et al., 2013 | Rectal only | Elective only | Before only | NR | ICGA | 16 | NR | Rob  100 | LAR  100 | NR | 75 | Clinical | 1 (6.3) | A/B/C  0/1/0 | NR |
|  |  |  |  |  | Control | 22 |  | Rob  100 | LAR  100 |  | 77.3 |  | 4 (18.1) | A/B/C  0/2/2 |  |
| Kim et al., 2017 | Rectal only | NR | Before and after | NR | ICGA | 310 | >700 cases of robotic rectal cancer operations | Rob  100 | LAR/ULAR  74.8/25.2 | NR | 54.8 | Clinical | 2 (0.6) | NR | 11 (3.5) |
|  |  |  |  |  | Control | 347 |  | Rob  100 | LAR/ULAR  66.3/33.7 |  | 34.6 |  | 18 (5.2) |  | 9 (2.6) |
| Wada et al., 2019 | Rectal only | Elective only | Before only | NR | ICGA | 48 | Board certified | Lap  100 | LAR  100 | NR | 0 | Clinical | 5 (10.4) | A/B/C  0/1/4 | NR |
|  |  |  |  |  | Control | 101 |  | Lap  100 | LAR  100 |  | 0 |  | 7 (6.9) | A/B/C  0/4/3 |  |
| Ris et al., 2018 | Multiple sites throughout colon and rectum | Elective only | Before and after | NR | ICGA | 504 | NR | Lap/O/Con  84.3/15.7/5.9 | RC/HAR/LAR/Other  28.4/37.9/17.9/15.9 | NR | NR | Clinical | 12 (2.4) | NR | NR |
|  |  |  |  |  | Control | 1173 |  | NR | NR |  |  |  | 68 (5.8) |  |  |
| Mizrahi et al., 2018 | Rectal only | Elective only | Before and after | NR | ICGA | 29 | NR | Lap/Con  100/0 | LAR  100 | 100 | 100 | Clinical | 0 (0) | A/B/C  0/0/0 | NR |
|  |  |  |  |  | Control | 30 |  | Lap/Con  100/3.3 | LAR  100 | 100 | 100 |  | 2 (6.7) | A/B/C  0/2/0 |  |
| Foo et al., 2020 | Left including rectal | Elective only | Before only | Yes | ICGA | 253 | NR | Lap or Rob/O/Con  93.7/6.3/4.2 | TME/Non-TME  50.2/49.8 | NR | 51.8 | Clinical | 9 (3.6) | A/B/C  2/3/4 | 5 (2) |
|  |  |  |  |  | Control | 253 |  | Lap or Rob/O/Con  88.9/11.1/4 | TME/Non-TME  54.5/45.5 |  | 50.6 |  | 20 (7.9) | A/B/C  5/10/5 | 6 (2.4) |
| Flores-Rodriguez et al., 2023 | Multiple sites throughout colon and rectum | Elective only | Before only | NR | ICGA | 280 | Senior surgeon or supervised trainee, no difference in anastomotic leak rate | Lap/O/Con  81/19/4.7 | RC/LC/AR  28.6/61.8/9.6 | NR | 0 | Clinical | 21 (7.5) | NR | NR |
|  |  |  |  |  | Control | 505 |  |  | RC/LC/AR  38.6/56.8/4.6 |  | 0 |  | 47 (9.3) |  |  |
| Baset et al., 2022 | Multiple sites throughout colon and rectum | NR | Before and after | NR | ICGA | 16 | NR | Lap  100 | RC/LC/SC/HAR/LAR  25/6.3/18.8/18.8/31.3 | NR | 31.3 | Clinical | 1 (6.3) | A/B/C  0/1/0 | NR |
|  |  |  |  |  | Control | 23 |  | Lap  100 | RC/LC/SC/HAR/LAR  30.4/17.4/13/21.7/17.4 |  | 17.4 |  | 2 (8.7) | A/B/C  0/1/1 |  |
| Brescia et al., 2018 | Multiple sites throughout colon and rectum | Elective only | Before and after | No | ICGA | 75 | Board-certified | Lap/Con  100/7.8 | RC/LC/AR/Other  29.7/30.8/28.6/11 | NR | NR | Clinical | 0 (0) | A/B/C  0/0/0 | NR |
|  |  |  |  |  | Control | 107 |  |  |  |  |  |  | 6 (5.6) | A/B/C  0/2/4 |  |
| Starker et al., 2017 | Multiple sites throughout colon and rectum | Elective and emergency | Before and after | NR | ICGA | 238 | NR | Lap/O  87/13 | NR | NR | 5.5 | Clinical | 2 (0.8) | NR | 0 (0) |
|  |  |  |  |  | Control | 109 |  | Lap/O  49.5/50.5 |  |  | 13.8 |  | 4 (3.7) |  | 2 (1.8) |
